# Deciphering macrophage differentiation and cell death dynamics in heart failure: a single-cell sequencing odyssey

**DOI:** 10.3389/fimmu.2025.1604226

**Published:** 2025-10-07

**Authors:** Jin Wei, Yao Sun, Bao-xi Qu, Xin-xin Duan, Li-hong Yan, Wei An, Kun-lun Yin, Shui-yun Wang, Yan-hai Meng, Lei Huang

**Affiliations:** ^1^ Ward General Practice, Tianjin Third Central Hospital, Tianjin, China; ^2^ Nankai University Affinity the Third Central Hospital, Tianjin, China; ^3^ Tianjin Key Laboratory of Extracorporeal Life Support for Critical Diseases, Tianjin, China; ^4^ Artificial Cell Engineering Technology Research Center, Tianjin, China; ^5^ Tianjin Institute of Hepatobiliary Disease, Tianjin, China; ^6^ State Key Laboratory of Cardiovascular Disease, Fuwai Hospital, National Center for Cardiovascular Diseases, Chinese Academy of Medical Sciences and Peking Union Medical College, Beijing, China; ^7^ Center of Laboratory Medicine, Fuwai Hospital, National Center for Cardiovascular Diseases, Chinese Academy of Medical Sciences and Peking Union Medical College, Beijing, China; ^8^ Department of Cardiovascular Surgery, Fuwai Hospital, National Center for Cardiovascular Diseases, Chinese Academy of Medical Sciences and Peking Union Medical College, Beijing, China; ^9^ Department of Surgical Intensive Care Unit, Fuwai Hospital, National Center for Cardiovascular Diseases, Chinese Academy of Medical Sciences and Peking Union Medical College, Beijing, China; ^10^ Heart Center, Tianjin Third Central Hospital, Tianjin, China

**Keywords:** macrophages, cardiomyopathy, single-cell RNA sequencing, anoikis, ferroptosis, cell death

## Abstract

**Aims:**

We hypothesize that specific macrophage differentiation trajectories in heart failure (HF) are coupled with subtype-specific and context-dependent engagement of programmed cell death (PCD) pathways, particularly ferroptosis and anoikis, which in turn influence disease progression and remodeling. HF is a progressive and heterogeneous clinical syndrome characterized by adverse immune remodeling, yet the precise contributions of macrophage heterogeneity, lineage dynamics, and PCD programs to its pathogenesis remain unclear. This study aimed to delineate, at single-cell resolution, the cellular and molecular landscape of cardiac macrophage subpopulations and their engagement with immunogenic cell death programs.

**Methods:**

We profiled human cardiac tissues from HF and non-failing donors using scRNA-seq from the SCP1303 dataset, initially comprising ~600,000 cells and reduced to ~120,000 high-quality cells from 18 samples after stringent quality control to retain biologically valid but metabolically distinct populations. Standardized cell-type annotation and pseudotime trajectory reconstruction were applied. Pathway activity was quantified using AUCell (primary) and GSVA (complementary) for cell death–related signatures. Integrated differential expression analysis, protein–protein interaction network mapping, and multi-algorithm feature selection (LASSO, SVM-RFE, Random Forest) were performed, and candidate biomarkers were validated using an independent bulk RNA-seq dataset (GSE57345).

**Results:**

Thirteen major cardiac cell types were identified, with macrophages showing the highest transcriptional heterogeneity. We resolved four macrophage subtypes and mapped bifurcating disease-associated differentiation trajectories, revealing distinct activation patterns of ferroptosis- and anoikis-related pathways. Ferroptosis-associated genes and anoikis-associated genes displayed subtype-specific enrichment and significant differential activation in HF. Pseudotime analysis demonstrated that suppression of ferroptosis and anoikis was linked to late-stage, HF-enriched macrophage states. Key biomarkers—including CD163, FPR1, and VSIG4—achieved robust diagnostic performance (AUC > 0.80) in discriminating HF phenotypes.

**Conclusions:**

This is the first study to integrate scRNA-seq, differentiation trajectory inference, and PCD pathway scoring to define the context-dependent engagement of ferroptosis and anoikis in macrophage subtypes in HF. The identification of subtype-specific biomarkers and functional states provides novel mechanistic insight and potential diagnostic and therapeutic targets, underscoring the value of high-resolution immune profiling for precision immunology in cardiovascular disease.

## Introduction

Heart failure (HF), resulting from diverse cardiac structural or functional aberrations, is a complex syndrome that disrupts ventricular filling and/or ejection. It is characterized by pulmonary and/or systemic congestion and insufficient blood perfusion to organs and tissues. HF is recognized as a major culprit for global morbidity and mortality, impacting more than 26 million people worldwide (1). Despite advancements in pharmacological interventions and device-based therapies, the prognosis of HF remains unpromising, with a 5-year survival rate of less than 50% (2). Therefore, understanding the cellular and molecular mechanisms underlying HF and identifying novel therapeutic targets and biomarkers remain urgent priorities.

In recent years, bulk RNA sequencing (bulk RNA-seq)numerous studies have utilized has been widely used to investigate transcriptomic changes in cardiac tissue under various disease conditions. However, bulk RNA-seq has intrinsic limitations, as it cannot resolve cell type-specific alterations or capture the functional diversity within a given lineage. Single-cell RNA sequencing (scRNA-seq) overcomes these limitations by enabling high -resolution and unbiased dissection of transcriptomic profiles at the individual cell level. This technology not only reveals the gene expression landscapes and functional states of distinct cell types, but also uncovers their dynamic transitions and interactions within the tissue microenvironment (3). scRNA-seq has been successfully applied to characterize cellular heterogeneity in cardiovascular diseases, including atherosclerosis, myocardial infarction, and cardiomyopathies, offering new mechanistic insights (4–6). However, despite the increasing recognition of macrophages as central players in HF pathogenesis, no study has systematically mapped their differentiation trajectories and programmed cell death (PCD) dynamics—particularly ferroptosis and anoikis—at single-cell resolution in human HF.

Ferroptosis and anoikis have been implicated in adverse cardiac remodeling and HF progression in preclinical and bulk-tissue studies (7, 8), but their cell type–specific regulation, temporal engagement along differentiation continua, and relationship to macrophage functional states remain undefined. This knowledge gap limits our ability to identify precise intervention points that could modulate maladaptive immune responses without impairing reparative processes.

Here, we address this gap by integrating scRNA-seq analysis, pseudotime trajectory inference, and cell death pathway activity scoring to test the central hypothesis that macrophage differentiation in HF involves subtype-specific and context-dependent engagement of ferroptosis and anoikis programs. We further hypothesize that these pathways converge with distinct transcriptional and functional phenotypes that can be exploited for diagnostic and therapeutic purposes. Our study is the first to (i) resolve HF-enriched macrophage subtypes and their bifurcating differentiation trajectories, (ii) link reduced ferroptosis and anoikis activity to late-stage, maladaptive macrophage states, and (iii) identify and validate high-performing biomarkers (e.g., CD163, FPR1, VSIG4) in independent cohorts, providing both mechanistic insight and translational potential.

In this study, we performed single-cell transcriptomic profiling of cardiac tissue from patients with dilated cardiomyopathy (DCM) or hypertrophic cardiomyopathy (HCM), —two major etiologies of HF—and from healthy donors. We compared cell composition and gene expression across conditions, with special focus on macrophages due to their key roles in HF pathogenesis and progression (9, 10). By linking differentiation trajectory analysis with immunogenic cell death pathway mapping, we offer a comprehensive framework to understand macrophage functional plasticity in HF and to inform the development of precision immunomodulatory strategies.

## Materials and methods

### Data sources and processing

scRNA-seq data were obtained from the publicly available SCP1303 dataset via the Single Cell Portal (https://singlecell.broadinstitute.org/single_cell), which originally comprised approximately 600,000 cells from all available samples. This dataset included 11 samples from DCM, 15 from HCM, and 16 from NF heart tissues. Following rigorous quality control (QC) filtering—removing cells with fewer than 200 detected genes, genes expressed in fewer than 50 cells, potential doublets, and cells with >10% mitochondrial gene content—we retained ~120,000 high-quality cells from 18 selected samples for downstream analysis. This filtering approach, which yielded roughly 20% of the original cells, is consistent with established best practices in scRNA-seq studies to ensure biological reliability and minimize technical artifacts. These samples maintained a balanced sex distribution, comprising 6 NF samples (3 males: P1702, P1549, P1678; 3 females: P1582, P1600, P1515), 6 HCM samples (3 males: P1422, P1462, P1722; 3 females: P1508, P1447, P1726), and 6 DCM samples (3 males: P1358, P1472, P1617; 3 females: P1437, P1304, P1300).

We downloaded bulk RNA sequencing data from the GSE57345 dataset, which was publicly available from the Gene Expression Omnibus (https://www.ncbi.nlm.nih.gov/geo/). This dataset contained 136 NF and 177 HF samples, with phenotypes including disease type (idiopathic DCM/ischemic/non-failing), sex, and age. It was subsequently used for validation of our scRNA-seq findings.

### Single-cell RNA sequencing and data analysis

The scRNA-seq data was subjected to a series of analytical steps on Seurat (v5.0.1), comprising data integration, normalization, scaling, dimensionality reduction, clustering, and differential expression analysis. Specifically, SCTransform method was used to normalize the data, and the Harmony algorithm was applied to integrate the data across various samples. For dimensionality reduction, we leveraged principal component analysis (PCA), while cell clusting was facilitated by the Louvain algorithm. To identify cluster-specific and disease-specific marker genes, we utilized the FindAllMarkers and the FindConservedMarkers functions respectively. Subsequently, we employed the ‘FindClusters’ function from the Seurat R package to perform cell clustering, and then proceeded to simplify the data using the ‘RunUMAP’ function. Visualization was achieved through t-SNE and UMAP. Cell type annotation was carried out using the SingleR package (version 1.4.1) based on the Human Primary Cell Atlas reference. For the identification of cell subpopulations, we implemented unsupervised graph-based clustering using Leiden algorithm. t−SNE was used to visualize the distribution of major cell types, highlighting relative abundances across cardiomyocytes, fibroblasts, endothelial cells, and other populations (see Results).

### Cell death analysis

Pathway activity was assessed using AUCell (primary) and ssGSEA (complementary) methods. AUCell (version 1.20.2) was selected as the primary approach because its rank-based algorithm is well suited for single-cell RNA-seq data with high sparsity, enabling robust detection of per-cell pathway activity even when total gene counts vary substantially. ssGSEA, implemented via the GSEABase (v 1.58.0) and GSVA (v 1.44.5) packages, was applied in parallel to provide a complementary, gene set–based perspective and to cross-validate AUCell-derived findings. Alternative methods such as GSVA (bulk transcriptome–oriented, potentially biased by variable detection rates at the single-cell level) and PROGENy (restricted to predefined downstream targets, potentially omitting relevant upstream regulators and context-specific genes) were considered but not used as primary tools given the characteristics of our dataset.

Based on curated gene sets from the Cell Death Signaling Database, enrichment scores for 12 programmed cell death pathways were calculated for each cell using the ssGSEA function. For targeted analysis of anoikis and ferroptosis in macrophages, activity scores were computed using AUCell on the basis of the corresponding gene sets from the Molecular Signatures Database (MSigDB). The ssGSEA- and AUCell-derived pathway activity scores were subsequently compared across cell types and disease groups using the Wilcoxon rank-sum test and the Kruskal–Wallis test, as appropriate.

Threshold calibration for AUCell scoring was performed to distinguish active versus inactive pathway states, and activation state mapping was visualized in UMAP space (see Results).

### Cell trajectory and pseudotime analysis

Cell trajectory and pseudotime analysis were conducted using the Monocle 3 package (version 1.3.4). The *preprocess_cds* function was adopted to filter out low-quality cells and genes, while the *reduce_dimension* function was applied to perform nonlinear dimensionality through the UMAP method. We used the *cluster_cells* function to group the cells according to their UMAP coordinates and utilized the *learn_graph* function to deduce the cell trajectory based on the minimum spanning tree algorithm. After that, the *order_cells* function was used to assign pseudotime values to each cell in accordance with their respect trajectories and the *differentialGeneTest* function was then engaged to identify genes that were differentially expressed along the pseudotime axis. Furthermore, the *BEAM* function was used to identify genes that were differentially expressed between branches of the trajectory. We performed functional enrichment and network analysis of the pseudotime-related genes using the *clusterProfiler* package (version 3.18.0) and the *STRINGdb* package (version 2.0.2). Lastly, regression analysis was executed on the pseudotime values and gene expression levels using the *fit_models* function in Monocle 3.

RNA velocity analysis was performed to infer the direction and magnitude of transcriptomic change across macrophage subtypes, and latent time mapping was integrated with pseudotime trajectories to identify early− and late−stage populations. AUCell scores were calculated along pseudotime to assess dynamic changes in cell death pathway activity.

### Differential analysis among macrophage subtypes

Gene differential expression analysis was conducted using edgeR (v_4.0.16). Low-expression genes and miRNAs were filtered out in more than 10% of the samples with counts per million less than or equal to 1 (cpm ≤ 1). The threshold for differential expression was set at an FDR less than 0.05 (FDR < 0.05) and a fold change greater than 1.5 (fold change> 1.5).

Subtype−specific marker genes were identified based on differential expression patterns and functional annotations, followed by functional enrichment and network analysis to characterize biological roles.

### Bulk RNA sequencing and data analysis

The bulk RNA sequencing data went through quality control, normalization, and differential expression analysis, facilitated by the DESeq2 package (version 1.42.1). We employed the limma package (version 3.58.1), coupled with the ComBat method, to perform batch effect correction. Furthermore, we used the edgeR package (version 4.0.16), along with the camera method, to perform gene set enrichment analysis. To distinguish key genes between HF and non-HF conditions, we utilized the ROC curve, principal component analysis, and Random Forest analysis within the MetaboAnalyst online tool (https://www.metaboanalyst.ca/).

Differentially expressed genes related to ferroptosis and anoikis were visualized in heatmaps and network diagrams, with subtype−specific distributions provided in **Supplementary Figures** (see Results).

### PPI networks and functional enrichment analysis

A Protein-Protein Interaction Network was constructed for the differentially expressed genes. Relevant genes were selected and used to develop the Protein-Protein Interaction Network with the assistance of the interaction gene retrieval tool, STRING (https://cn.string-db.org/cgi/input?sessionId=bgCn09WJHNWX&input_page_show_search=on), and Cytoscape (v_3.8.2). Additionally, we conducted Kyoto Encyclopedia of Genes and Genomes pathway analysis within the KOBAS database. The significance of the Kyoto Encyclopedia of Genes and Genomes analysis results was evaluated using the p-value obtained from the hypergeometric test.

### Ethics statement

Ethical approval was not required for this study since it involves the analysis of publicly available datasets. These datasets—SCP1303 (Single Cell Portal) and GSE57345 (Gene Expression Omnibus)—had been previously collected and anonymized in accordance with ethical guidelines of the 1975 Declaration of Helsinki, as revised in 2000. All data utilized in this study comply with the ethical standards of the repository source and institutional guidelines.

## Results

### Single-cell sequencing reveals cellular diversity in the failing heart

**Figure 1:**
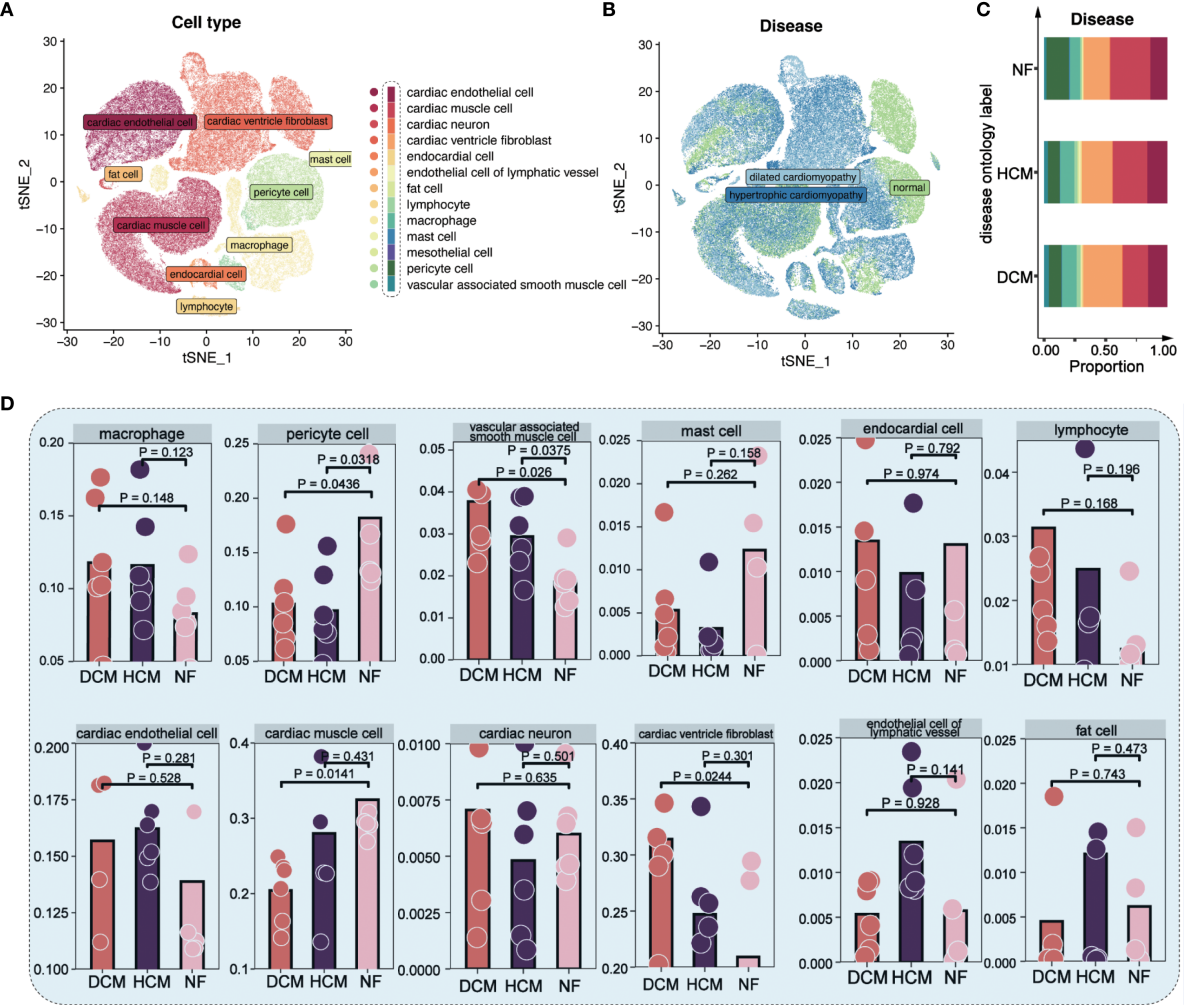
Single-cell analysis in 18 cardiomyopathy samples. **(A)** t−SNE plot showing 13 major cardiac cell types. Each color represents a distinct cell type as annotated in the legend; colors are categorical and do not indicate quantitative values. **(B)** t−SNE plot of cells from NF, HCM, and DCM patients. Each color corresponds to a disease group (NF = non−failing, HCM = hypertrophic cardiomyopathy, DCM = dilated cardiomyopathy); colors are categorical. **(C)** Proportions of each cell type in NF, HCM, and DCM samples. Colors denote distinct cell types, consistent with the scheme in **Figure 1A**. **(D)** Distribution of 12 major cell types across NF, HCM, and DCM samples.

We integrated and clustered scRNA-seq data from the SCP1303 dataset, retaining ~120,000 high-quality cells after QC filtering (see Methods), and identifying 13 major cell types using marker gene expression and the Human Primary Cell Atlas. These cell types are annotated in **Figure 1A**. t-distributed Stochastic Neighbor Embedding (t-SNE) visualization showed cardiomyocytes as the most abundant cell type, followed by fibroblasts and endothelial cells (**Figures 1A, B**). Cell-type proportions across NF, HCM, and DCM are shown in **Figure 1C**. Non-failing (NF) samples had a higher proportion of cardiomyocytes and pericytes, whereas DCM samples showed more ventricle fibroblasts (**Supplementary Figure S1**, **Figure 1D**).

### Macrophage diversity and distinct cell death pathways in HF

**Figure 2:**
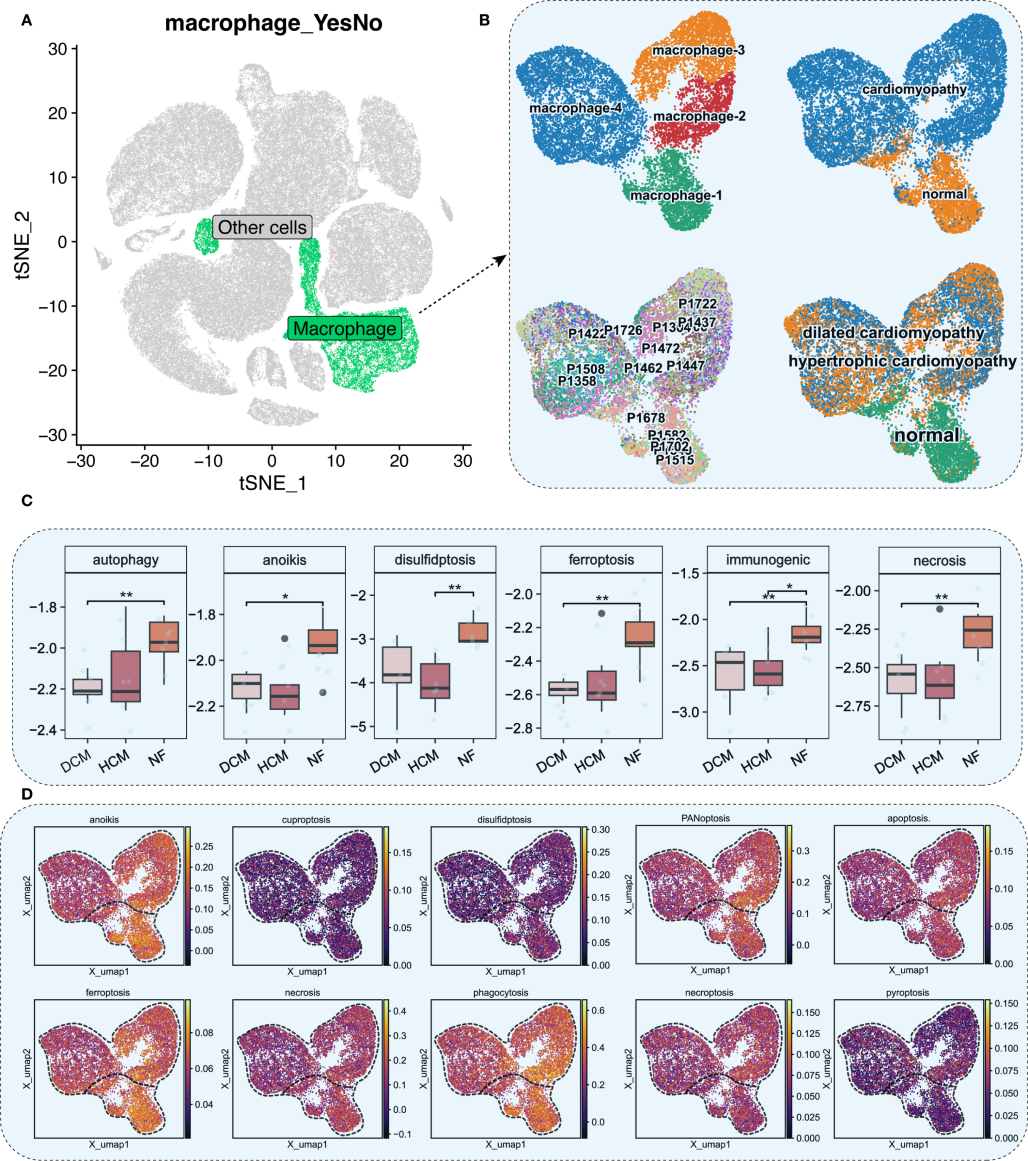
Macrophage analysis and cell death scoring. **(A)** t−SNE plot with macrophages in green. **(B)** UMAP plots of macrophage subpopulations across NF, HCM, and DCM samples. Each color represents a distinct macrophage subtype; colors are categorical. **(C)** ssGSEA scores for six cell death pathways in macrophages (*P*<0.05: *, *P*<0.01: **). **(D)** UMAP plot of ssGSEA−derived activity scores for 10 cell death pathways. Color gradient from dark blue (low activity) to bright yellow (high activity) represents relative pathway activity per cell.

**Figure 3:**
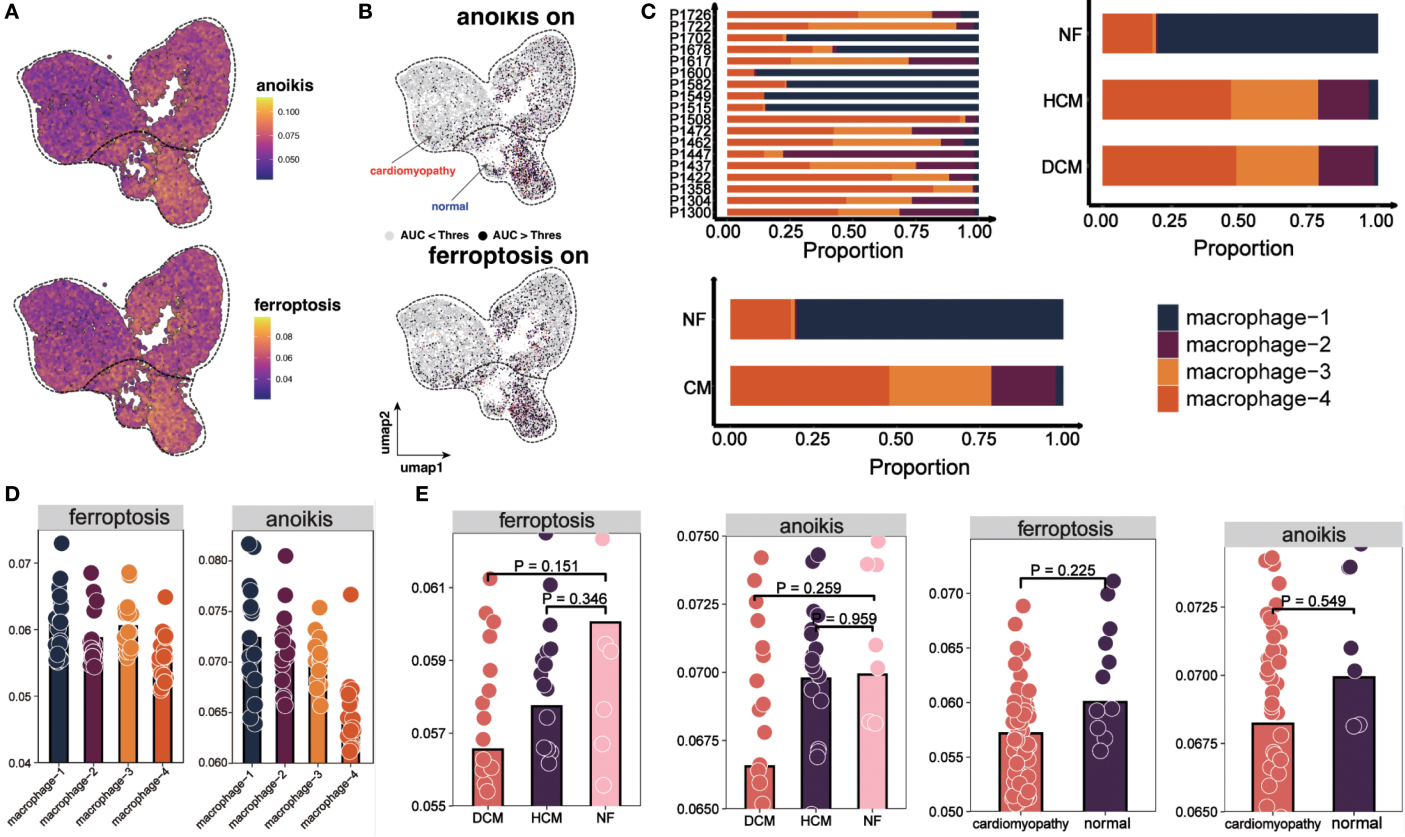
Distribution and cell death states of macrophages. **(A)** UMAP plot showing AUCell scores for ferroptosis and anoikis, lighter colors indicate higher scores. **(B)** UMAP plots showing activation states for anoikis (top) and ferroptosis (bottom). Cells are colored by activation status derived from per−cell AUCell AUC values: black = active (AUC > threshold), gray = inactive (AUC < threshold). The overlaid red (“cardiomyopathy”) and blue (“normal”) labels denote disease−associated regions and do not indicate cell−level color coding. **(C)** Proportions of four macrophage subpopulations across 18 patients, NF, HCM, and DCM samples. **(D)** AUCell scores for ferroptosis and anoikis in macrophage subpopulations. Bar height indicates median pathway activity score. **(E)** AUCell scores for ferroptosis and anoikis in NF, HCM, DCM, and combined CM samples. Bar height indicates median pathway activity score.

We focused on macrophages, key immune cells in HF. Reclustering identified four macrophage subtypes (macrophage-1 to -4) based on expression patterns and functional annotations (**Figures 2A, B**). Single-sample Gene Set Enrichment Analysis (ssGSEA) revealed distinct expression patterns in autophagy, anoikis, ferroptosis, and necrosis between HF and NF macrophages (**Figures 2C, D**, **Supplementary Figure S2**). AUCell scores revealed diminished anoikis and ferroptosis in HF samples (**Figure 3A**), with threshold calibration in **Supplementary Figure S3** and activation state mapping in **Figure 3B**. Active anoikis- and ferroptosis-positive macrophages were predominantly macrophage-1 and -2, while inactive ones were macrophage-3 and -4.

Our analysis revealed distinct distribution patterns of macrophage subtypes across myocardial samples: macrophage-1 and macrophage-2 were enriched in NF, whereas macrophage-3 and macrophage-4 were more abundant in HCM and DCM (**Figure 3C**). Consistently, macrophages active in anoikis and ferroptosis were mainly found in macrophage-1 and macrophage-2, whereas inactive macrophages localized to macrophage-3 and macrophage-4 (**Figure 3D**). Furthermore, AUCell scoring demonstrated that anoikis and ferroptosis activity levels were normal in NF but significantly diminished in HF (**Figure 3E**).

### Macrophage differentiation trajectory and pseudotime-related gene expression in HF

**Figure 4:**
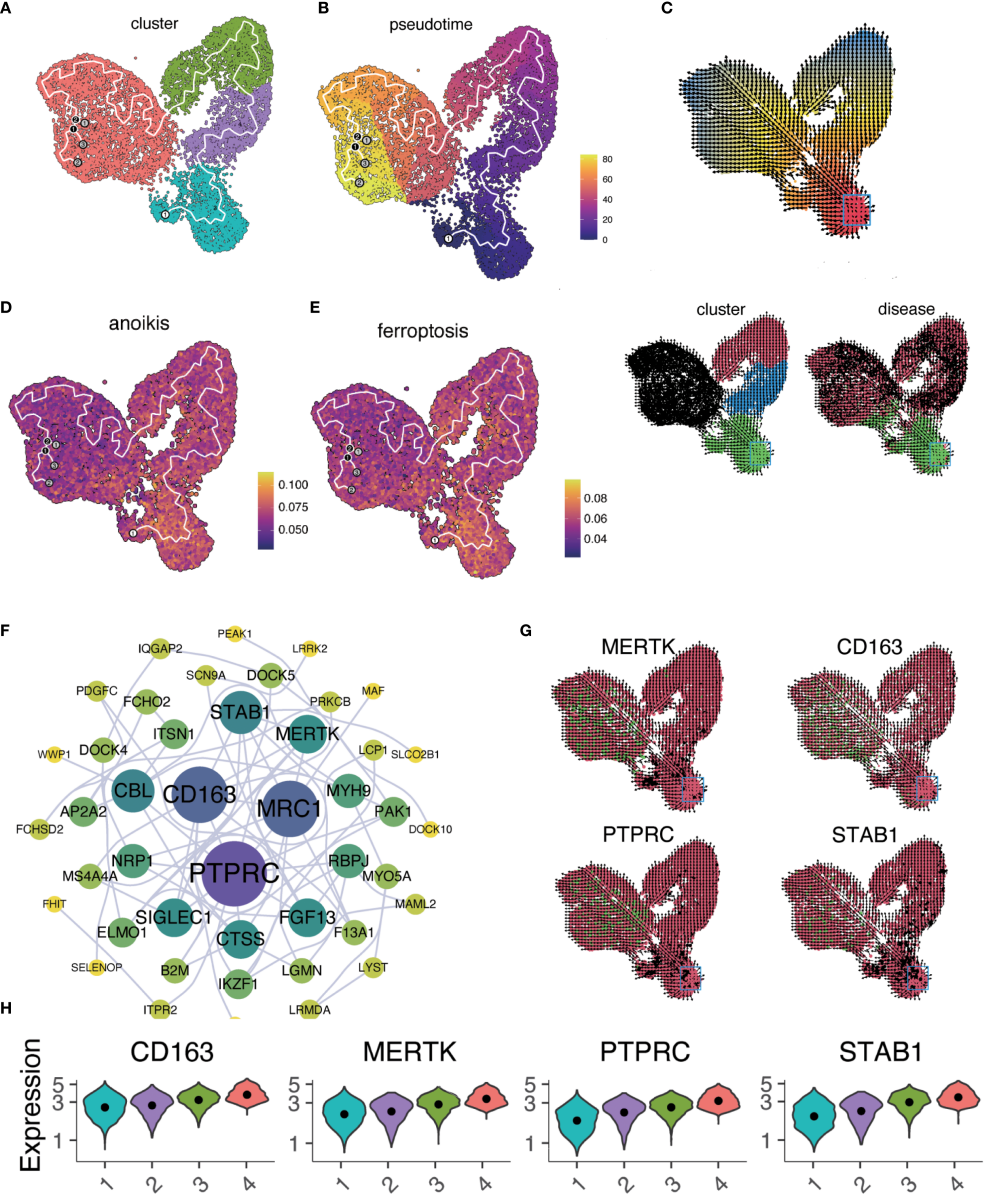
Macrophage trajectory analysis and RNA velocity. **(A)** UMAP plot of pseudotime trajectory for macrophage subtypes using Monocle 3. **(B)** Pseudotime sequence analysis with cells colored by pseudotemporal orders. Gradient from dark purple (early) to bright yellow (late). **(C)** RNA velocity analysis of macrophage differentiation states visualized in three panels. Arrows indicate the inferred direction and relative speed of transcriptomic change. Top: Cells colored by latent time inferred from RNA velocity (continuous gradient from early [red] to late [blue] states). Bottom left: Same embedding colored by macrophage subclusters (categorical; macrophage 1 to macrophage 4). Bottom right: Same embedding colored by disease status (categorical). **(D, E)**. AUCell scores for anoikis **(D)** and ferroptosis **(E)** mapped along the pseudotime trajectory. In both panels, the color gradient from dark blue (low activity) to bright yellow (high activity) denotes relative pathway activity at the single−cell level. **(F)** Interaction network of genes from regression analysis. Node color indicates pathway association: purple = anoikis−related, blue = ferroptosis−related, red = both; node size reflects degree centrality. Notable interactions include PTPRC and CD163, which co−localize within the network despite their traditionally opposing functional associations. This co−expression pattern may indicate transitional macrophage states with both inflammatory signaling potential and reparative capacity. **(G)** Expression patterns of key genes (*MERTK, CD163, PTPRC, STAB*1) across the tissue section. Tiles are colored by normalized expression for the indicated gene: red denotes lower expression, green denotes higher expression. Color scaling is applied per panel. **(H)** Time−series regression analysis of key gene expression changes across macrophage clusters.

Trajectory analysis (Monocle 3) revealed a bifurcated path from macrophage-1 to macrophage-4 (**Figures 4A, B**). RNA velocity indicated greater vectior magnitudes in macrophage-3 and macrophage-4 (**Figure 4C**, bottom left). The latent time map (**Figure 4C**, top) and integration with pseudotime analysis placed M3 and M4 at later stages of differentiation originating from M1. Reduced anoikis and ferroptosis in these late-stage subsets suggests an adaptive survival advantage in the pro-inflammatory, stress-rich failing myocardium. Key pseudotime-correlated genes (e.g., *FRMD4A*, *CD163*, *NEAT1*) are shown in **Supplementary Figure S4**. AUCell scores along the trajectory confirmed reduced cell death pathways activation in differentiated macrophages (**Figures 4D, E**). As pseudotime progressed, increases in cell counts, gene counts, and label diversity signified rising complexity within differentiated macrophages (**Supplementary Figure S5**). Pseudotime–gene regression and functional enrichment summaries are provided in **Supplementary Figures S6A, B**. Gene interaction network (*PTPRC, STAB1, CD163, MERTK*) are shown in **Figure 4F**, progressive increases in *MERTK, PTPRC, CD163, STAB1* expression with advancing HF are shown in **Figures 4G, H**. The expression and distribution of significantly downregulated key genes in diseased tissues are shown in **Supplementary Figure S7**.

### Functional analysis of macrophage subtypes

**Figure 5:**
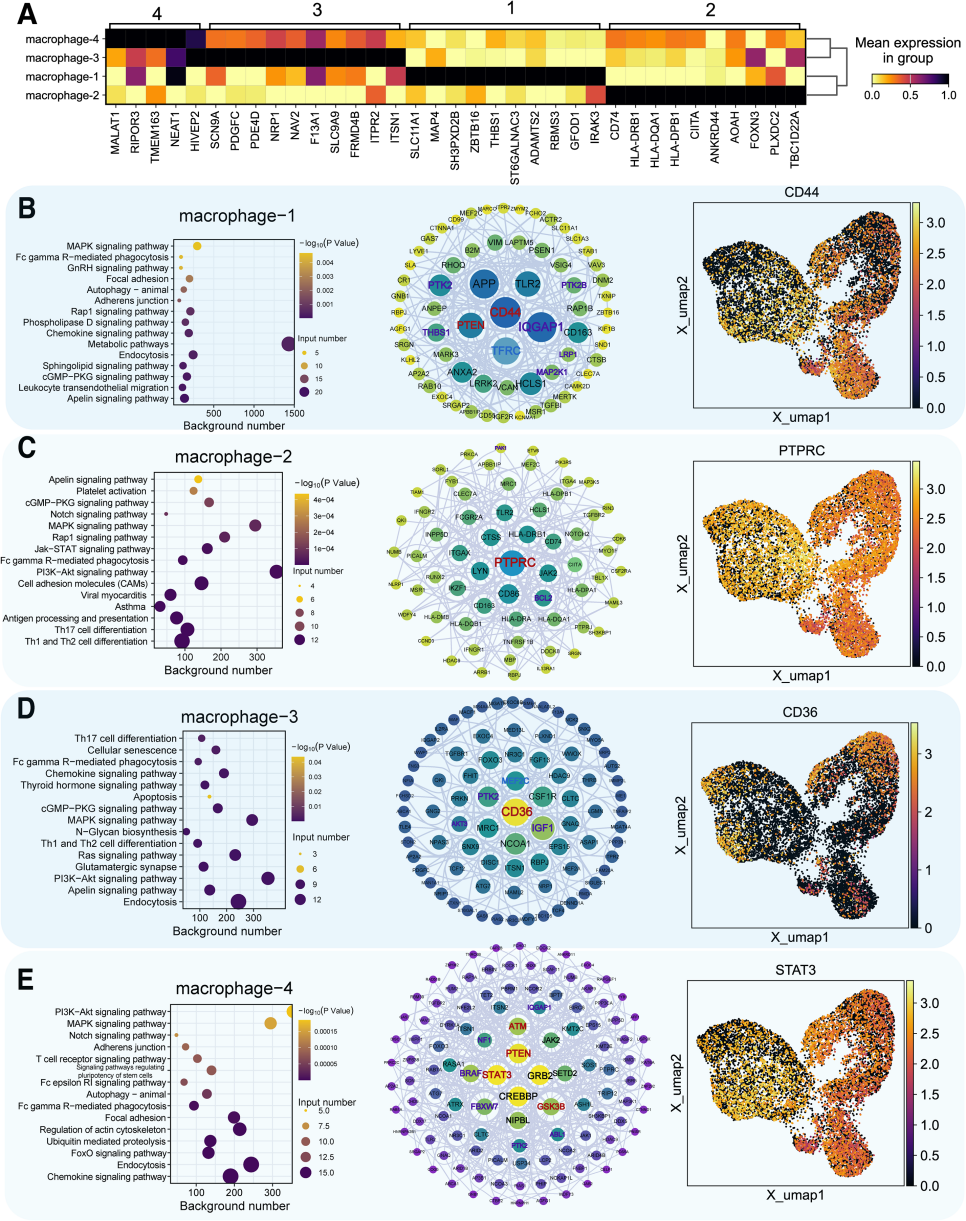
Functional analysis of key marker genes in macrophage subpopulations. **(A)** Marker gene identification in four macrophage clusters, darker colors indicate higher expression. **(B–E)** For each macrophage subtype (type 1–4): Left panel = functional enrichment bubble plot, where bubble color encodes statistical significance (–log_10_ P value, continuous gradient) and bubble size represents the number of input genes associated with each pathway. Middle panel = gene interaction network (node color indicates pathway association: purple = anoikis−related, blue = ferroptosis−related, red = both; node size reflects degree centrality). Right panel = UMAP plot of marker gene distribution (color gradient from dark blue = low expression to bright yellow = high expression).

We identified key marker genes for each macrophage subtype from differential expression patterns and functional annotations. Functional enrichment and network analysis revealed distinct biological roles: macrophage−1 (metabolic pathways), macrophage−2 (immune responses, PI3K–Akt signaling), macrophage−3 (endocytosis), macrophage−4 (chemokine signaling) (**Figures 5A–E**). In HF, PI3K–Akt activation in macrophage-2 may support reparative processes but could also promote fibrosis under persistent inflammation.

### Gene expression differences in macrophages from healthy and cardiomyopathy patients

**Figure 6:**
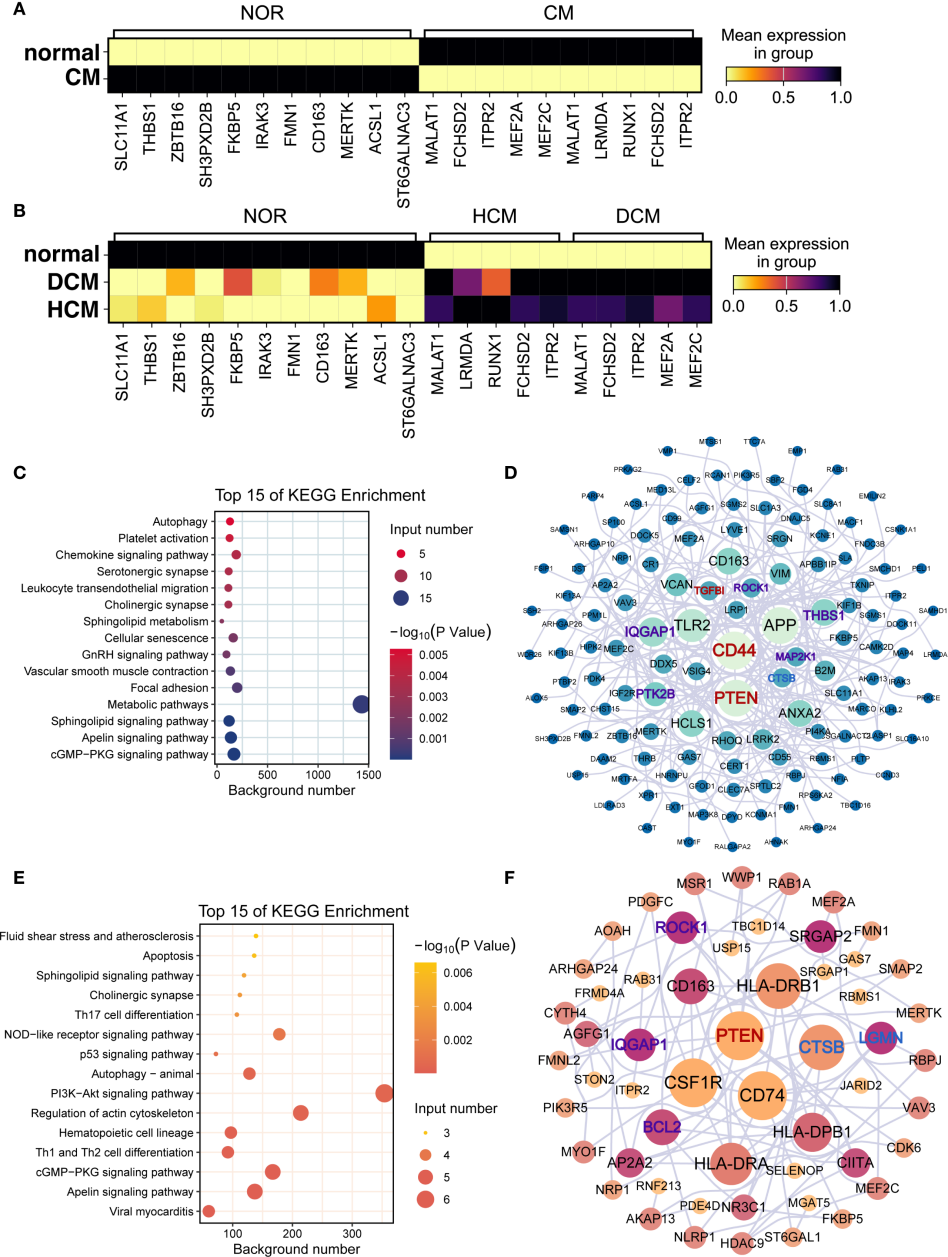
Functional differences and key differentially expressed genes in macrophages. **(A, B)** Marker gene expression in macrophages from **(A)** normal vs. cardiomyopathy samples and **(B)** NF, DCM, and HCM samples. In both panels, color gradient from light yellow (low value) through red (intermediate value) to black (high value) represents mean expression in group. **(C–F)** Functional enrichment and interaction networks of marker genes in normal **(C, D)** and cardiomyopathy **(E, F)** samples. **(C, E)** Bubble plots of enriched KEGG pathways. Bubble color encodes the –log10(p) value (continuous scale), and bubble size represents the number of genes associated with each pathway. **(D, F)** Protein–protein interaction networks of marker genes. Node color indicates pathway association (purple = anoikis−related, blue = ferroptosis−related, red = both), and node size reflects degree centrality.

Differential expression analysis revealed distinct patterns for *PTPRC*, *STAB1*, *CD163*, and *MERTK* (**Figures 6A, B**). Functional enrichment showed ‘Metabolic pathway’ predominance in normal samples (**Figure 6C**) and ‘PI3K-Akt signaling’ in HF (**Figure 6E**). In healthy hearts, *CD44* and *PTEN* were linked to ferroptosis and anoikis (**Figure 6D**), while in HF, *CSF1R*, *CD74*, and *PTEN* were central (**Figure 6F**). Differentially expressed genes related to ferroptosis and anoikis in healthy and diseased groups are detailed in **Supplementary Figures S8**, **S9**. Subtype-specific marker gene distributions for macrophage-1, macrophage-2, and macrophage-3 are provided in **Supplementary Figures S10**-**S12**.

### Macrophage terminal differentiation and key gene identification

**Figure 7:**
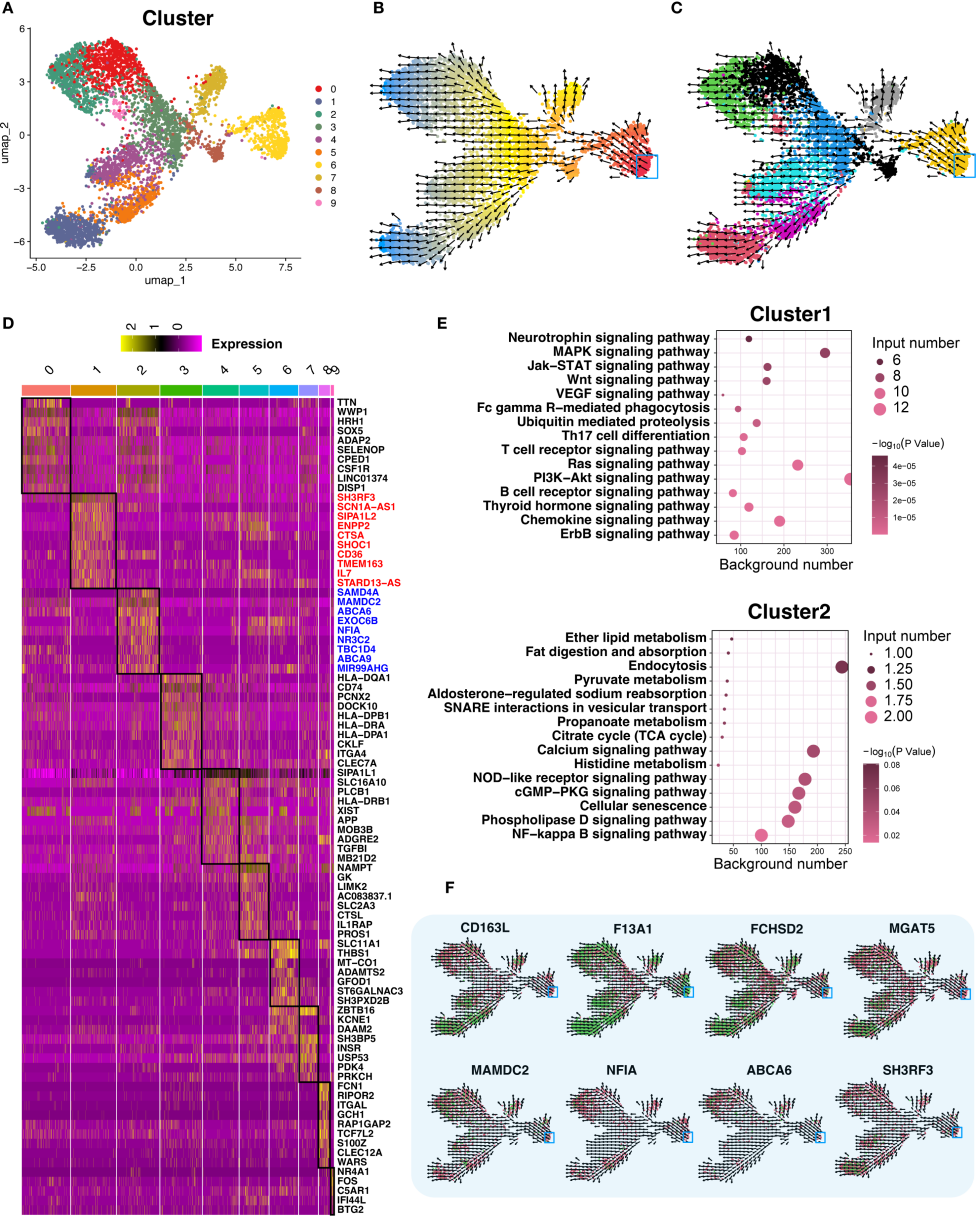
Trajectory analysis of terminally differentiated Macrophage−4. **(A)** UMAP re−clustering of Macrophage−4 into 10 subclusters; each color denotes a distinct subcluster (categorical). **(B)** RNA velocity analysis of Macrophage−4. Cells are colored by latent time inferred from RNA velocity (continuous gradient from early to late states), with the start point in red and the endpoint in blue marking the trajectory extremes. Arrows indicate the inferred direction and relative speed of transcriptomic change. **(C)** RNA velocity analysis across all 10 subclusters, colored by subcluster identity (categorical) using a palette different from panel **(A)**. Arrows indicate inferred direction and speed. **(D)** Differential expression heatmap of the 10 subclusters, ordered as in panel **(A)**; within the heatmap, yellow indicates higher expression and darker shades indicate lower expression. Black boxes highlight the regions of interest corresponding to each subcluster’s differentially expressed genes. A dedicated column on the right lists representative marker genes for these regions. **(E)** Bubble plots of functional enrichment for clusters 1 and 2; bubble color encodes statistical significance (–log10 *P* value, continuous gradient), and bubble size reflects the number of associated genes. **(F)** Expression and RNA velocity analysis of marker genes in clusters 1 and 2; color gradient from green (high expression) to red (low expression) represents normalized expression level, with arrows indicating inferred differentiation direction.

We mapped the developmental trajectory of macrophage-4, the most prevalent subtype, into 10 subclusters (macrophage-4.1 to -4.10) (**Figure 7A**). Clusters 1 (DCM) and 2 (HCM) marked terminal differentiation stages. RNA velocity supported these trajectories (**Figures 7B, C**). Key genes are shown in heatmaps (**Figure 7D**), with functional enrichment and network analyses in **Figure 7E**, and gene expression trajectories in **Figure 7F**.

### Validation of macrophage marker genes and prognostic power

**Figure 8:**
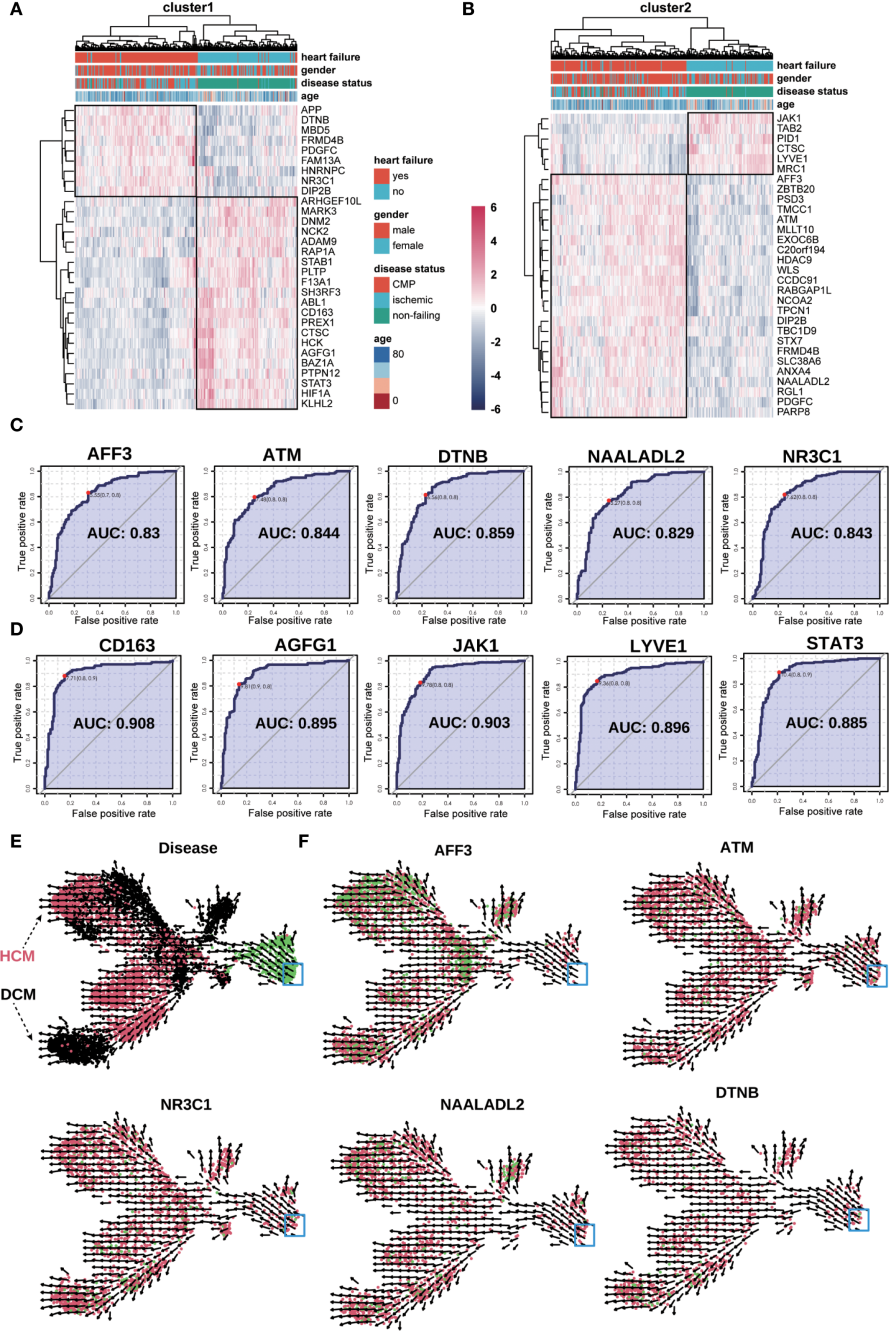
Validation of key genes in the bulk−seq dataset (GSE57345). **(A, B)** Heatmap validation of marker genes from Macrophage−4 clusters 1 **(A)** and 2 **(B)**. The color scale denotes normalized expression (dark blue = low, red = high); columns are grouped by clinical category (e.g., NF, DCM, HCM). **(C, D)** ROC curve validation of selected upregulated **(C)** and downregulated **(D)** marker genes from clusters 1 and 2. Each curve represents one gene, with the corresponding AUC value indicated. **(E)** UMAP plot of RNA velocity analysis overlaid with disease classification, illustrating overall differentiation trajectories of the marker genes. Arrows indicate the inferred direction and relative speed of transcriptomic change, with color denoting expression level (green = high, red = intermediate, black = low). **(F)** UMAP plots showing expression and RNA velocity of selected marker genes (*NR3C1, ATM, NAALADL2, DTNB, AFF3*) in clusters 1 and 2. Color gradient from red (low expression) to green (high expression) represents normalized expression level, with arrows indicating inferred differentiation direction; blue boxes highlight regions of interest.

Using dataset GSE57345, we validated key marker genes for HF diagnosis (**Figures 8A, B**). Upregulated and downregulated genes are shown in **Figures 8C, D**. Receiver operating characteristic (ROC) curves indicated AUC > 0.8. UMAP and RNA velocity confirmed differentiation pathways and marker gene dynamics (**Figures 8E, F**).

### Multigenic model validation in HF

Random forest algorithm identified the top 20 genetic features distinguishing HF from non-failure groups; principal component analysis demonstrated diagnostic efficiency in **Supplementary Figures S13A, B**). ROC analysis for a 10-gene model showed robust predictive value (**Supplementary Figures S13C–E**).

## Discussion

Understanding how regulatory changes specific to cell lineages occur under disease conditions is crucial for effective drug development. This study used single-cell transcriptomics to analyze HF in patients with DCM or HCM. By comparing HF samples to healthy donors, we aimed to uncover novel disease insights. Our focus was on macrophages, key immune cells in HF pathogenesis and progression. We revealed macrophage evolutionary trajectories and their links to anoikis and ferroptosis, two modes of programmed cell death. Key genes differentially expressed in macrophages across HCM, DCM, and non-failing samples were identified, providing a comprehensive view of HF’s cellular and transcriptomic landscape. These findings have significant implications for the diagnosis, prognosis, and treatment of HF and other cardiovascular diseases.

### Macrophage differentiation and HF progression

Our scRNA-seq analysis of approximately 120–000 cells identified 13 distinct cell types, focusing on macrophages due to their critical roles in inflammation and tissue remodeling. Macrophages, which clear apoptotic cells and modulate inflammation, play diverse roles in cardiac health (11, 12). Pseudotime and RNA velocity analyses revealed detailed evolutionary trajectories and functional specializations of macrophage subtypes in HF. Understanding these subtypes helps identify cellular states contributing to HF progression, offering potential therapeutic targets.

Recent studies highlight the complexity of macrophage subtypes in cardiovascular diseases. For instance, Liu et al. identified eight immune subpopulations, including five macrophage subtypes, in HCM patients, underscoring altered macrophage dynamics (13). Another study found ten macrophage subsets involved in tissue repair and immune regulation (14). Consistent with these findings, we identified four macrophage subtypes and further subdivided macrophage-4 into ten subclusters, demonstrating macrophage specialization in response to different pathological states.

Our results suggest that macrophage transcriptomic states are continuous rather than discrete, with a proinflammatory FCN1-high subpopulation prevalent in diseased conditions. These findings align with studies (15, 16). Highlighting macrophage heterogeneity’s role in maintaining tissue homeostasis and responding to physiological changes.

### Distinct cell death pathways

Our analysis revealed significant differences in macrophage-mediated cell death mechanisms between HF and normal heart samples, particularly in autophagy, anoikis, ferroptosis, and necrosis. Reduced activities in anoikis and ferroptosis in HF samples suggest macrophage dysfunction is closely linked to HF pathophysiology. Macrophages in HF not only modulate immune responses but also engage in a complex network of cell death and repair mechanisms (11, 12). Studies indicate that HF macrophages have reduced autophagy-related gene expression, leading to waste accumulation and increased apoptosis, exacerbating cardiac decline (17, 18).

Macrophages promote myocardial repair by secreting cytokines, a process impaired in HF patients (19, 20). Altered macrophage polarization in HF affects functionality, providing insights into potential therapeutic interventions (21). Ferroptosis, characterized by excessive iron accumulation, plays a critical role in cardiac injury (22, 23). Reduced ferroptosis in HF samples, likely due to abnormal iron metabolism regulation, impacts heart stability. In the context of DCM and HCM, ferroptosis in macrophages may amplify local inflammation and oxidative stress through the release of damage−associated molecular patterns and pro−inflammatory mediators, thereby exacerbating cardiomyocyte injury and promoting maladaptive ventricular remodeling (24, 25). Anoikis, triggered by the loss of cell–matrix interactions, may impair the retention and reparative capacity of macrophages within the myocardial interstitium, disrupting extracellular matrix turnover and favoring pathological fibrosis (25, 26). Together, these death pathways could shift macrophage populations toward pro−fibrotic and pro−inflammatory states, creating a feed−forward loop that accelerates adverse remodeling in both disease phenotypes. While our study is primarily computational, these mechanistic links are supported by prior experimental evidence in cardiovascular disease models and warrant targeted in−vitro and *in−vivo* validation in future work (24–26).

The observed heterogeneity in cell death pathways underscores the necessity for targeted therapeutic strategies addressing specific macrophage subtypes and their unique roles in disease progression. Recent studies have further elucidated the role of macrophage-mediated cell death in cardiovascular diseases. For instance, a study highlighted the multiple roles of cardiac macrophages in heart homeostasis and failure, emphasizing their importance in immune defense, apoptotic cell clearance, and regulation of electrical conduction and arterial tone (27). Our findings on distinct cell death pathways offer a nuanced understanding of macrophage functions in HF, suggesting targeted therapeutic strategies for specific macrophage subtypes could improve treatments.

### Macrophage metabolic reprogramming and adaptive responses in HF

To further investigate the role of macrophages in HF, recent metabolomics studies have identified significant changes in macrophage metabolic pathways in HF patients, particularly enhanced glycolytic activity and reduced oxidative phosphorylation (13, 28). This metabolic shift indicates macrophage dysfunction, contributing to disease progression. Our study uses pseudotime and RNA velocity analyses to map the developmental trajectories of macrophages in HF. We found that macrophage-1 subtypes are initially enriched in metabolic processes vital for cellular energy homeostasis. As HF progresses, macrophage differentiation shifts towards pathways crucial for cell survival and proliferation, especially the PI3K-Akt signaling pathway. This transition underscores macrophages’ adaptive response to the failing heart’s demands, emphasizing their roles in immune responses and tissue remodeling.

The PI3K-Akt pathway is fundamental for protecting cells against stress-induced apoptosis, promoting cell proliferation, and enhancing survival under adverse conditions (29). Dysregulation of this pathway has been implicated in HF due to its role in adverse cardiac remodeling and apoptosis (13). Our identification of the PI3K-Akt pathway as central to macrophage differentiation highlights its crucial role in maintaining cardiovascular function and its potential as a therapeutic target. In our dataset, both M2 (NF-enriched) and M4 (HF-enriched) macrophage subsets exhibited PI3K–Akt activation; however, the downstream consequences appear context-dependent: in M2 cells, activation supports anti-inflammatory polarization, phagocytosis, and tissue repair within relatively stable microenvironments, contributing to the resolution of injury. In contrast, within M4 cells, a pro-inflammatory cytokine milieu (e.g., TNF-α, IL-6, IFN-γ) redirects PI3K–Akt signaling toward metabolic reprogramming, sustained inflammatory output, and prolonged survival of maladaptive macrophage phenotypes. While such activation in M2 macrophages may be beneficial in acute injury resolution, its persistence in M4 subsets could maladaptively drive fibroblast activation, extracellular matrix deposition, and profibrotic remodeling. This dual potential underscores both the therapeutic challenge and opportunity in modulating PI3K–Akt signaling with cell state– and microenvironment–specific precision.

By engaging alternative survival and proliferation pathways under stress, these macrophage subsets can maintain their presence and functionality within the failing heart, thereby sustaining immune surveillance and contributing to tissue remodeling. This adaptive plasticity exemplifies the dynamic nature of macrophages in HF, and understanding these state transitions offers strategic entry points for interventions aimed at reprogramming macrophage functions toward reparative rather than pathological outcomes.

### Key genetic markers and macrophage differentiation in HF

Our pseudotime and RNA velocity analyses mapped the developmental trajectories of macrophages in HF, identifying key genes such as *PTPRC, STAB1, CD163*, and *MERTK*. These genes are essential for understanding macrophage differentiation and their roles in HF progression. *PTPRC* and *STAB1* are involved in immune regulation, while *CD163* and *MERTK* are associated with anti-inflammatory and tissue repair processes. Interestingly, co-expression of PTPRC (a pro-inflammatory pan-leukocyte receptor tyrosine phosphatase) and CD163 (an anti-inflammatory scavenger receptor) was observed in a subset of macrophages positioned at intermediate points along the differentiation trajectory. Literature and network analysis suggest that such dual-expression states may confer phenotypic plasticity, enabling macrophages to rapidly toggle between inflammatory and reparative functions in response to shifting microenvironmental signals. In HF, this hybrid phenotype may be critical for navigating the alternating phases of injury and repair characteristic of the failing myocardium. The progressive increase in expression levels of these genes across macrophage subtypes suggests their involvement in the maturation process and functional specialization in HF.

Recent studies highlight the importance of gene expression trajectories in understanding macrophage specialization. MERTK is crucial for efferocytosis, clearing apoptotic cells and preventing secondary necrosis and inflammation (30). Elevated *MERTK* expression in later macrophage subtypes underscores its role in inflammation resolution. PTPRC (CD45) is significant in regulating immune cell signaling and is essential for T-cell receptor signaling and cytokine production, impacting the immune response during cardiac stress (31, 32). Increased *PTPRC* expression across macrophage subtypes indicates its role in immune response and potential as a biomarker for HF progression. CD163, a marker of anti-inflammatory macrophages, is involved in clearing hemoglobin-haptoglobin complexes (33). Research indicates that CD163 expression is upregulated in response to inflammatory stimuli, and its soluble form serves as a biomarker for various inflammatory diseases, including cardiovascular conditions (34). Our observation of increased CD163 expression in later macrophage subtypes is consistent with its role in modulating inflammation, highlighting its potential as a therapeutic target.

### Prognostic and diagnostic implications of multigenic models

Validating key marker genes using the GSE57345 dataset demonstrated robust predictive accuracy for diagnosing HF phenotypes, specifically HCM and DCM. Integrating macrophage differentiation clusters into multigenic predictive models enhances diagnostic efficiency and provides insights into HF’s molecular mechanisms. ROC curve analysis indicated strong diagnostic capabilities with AUC values exceeding 0.8, underscoring these markers’ clinical utility. To reduce the risk of overfitting in ROC analysis, we applied k−fold cross−validation (k = 10) when estimating AUC values for individual genes. While this approach provided more robust performance estimates, we acknowledge that the absence of validation in an independent external cohort remains a limitation, and future studies will aim to confirm these findings in datasets from other centers. Our multigenic model, with genes like *CD44*, *PTEN*, and *CSF1R*, enhances early diagnosis and patient stratification in HF.

The high predictive accuracy of these genetic markers suggests their effective use in clinical settings for diagnosing HCM and DCM. For example, CD44 is implicated in cell adhesion and migration, critical in cardiac remodeling and HF (35). PTEN, a key regulator of the PI3K-Akt signaling pathway, is involved in cell survival and metabolism, with dysregulation linked to adverse cardiac remodeling and HF (36). CSF1R, a receptor for macrophage colony-stimulating factor, plays a crucial role in macrophage differentiation and inflammation (37). These studies corroborate our findings, supporting the enhancement of diagnostic accuracy by incorporating these genes into predictive models.

Integrating macrophage differentiation clusters into multigenic predictive models enhances robustness and reliability. By incorporating key genetic markers indicative of specific macrophage states, we develop more accurate and clinically relevant diagnostic tools. Upregulation of *CD44*, *PTEN*, and *CSF1R* in specific macrophage subtypes correlates with increased disease severity and poorer outcomes in HCM and DCM, serving as valuable indicators for early diagnosis and targeted treatment strategies.

## Conclusions and future directions

Macrophages play a complex and multifaceted role in HF, involving cell death, metabolic reprogramming, and cell-cell interactions. Our study offers a detailed characterization of macrophage diversity and their molecular mechanisms in HF. Identifying and validating key genetic markers as diagnostic tools underscore the clinical potential of these findings. This research enhances our understanding of macrophage biology in cardiovascular diseases and opens new avenues for targeted therapies and personalized medicine.

Future research should validate these findings in larger cohorts and explore targeting specific macrophage subtypes. Longitudinal studies could provide insights into dynamic changes in macrophage populations and their impact on disease progression. Additionally, focusing on macrophages’ multifaceted roles and regulatory mechanisms in HF could lead to novel therapeutic strategies, such as gene editing or pharmacological interventions targeting macrophage metabolic pathways, to improve HF prognosis.

## Data Availability

All data analyzed in this study are publicly available. Single-cell RNA sequencing data were obtained from SCP1303 via the Single Cell Portal (https://singlecell.broadinstitute.org/single_cell), and bulk RNA sequencing data were obtained from GSE57345 via the Gene Expression Omnibus (https://www.ncbi.nlm.nih.gov/geo/).
