# Supplementary figures and images for: Deciphering macrophage differentiation and cell death dynamics in heart failure: a single-cell sequencing odyssey
Source: Front Immunol. 2025 Oct 7;16:1604226. doi: 10.3389/fimmu.2025.1604226 (PMC12537381; doi:10.3389/fimmu.2025.1604226)

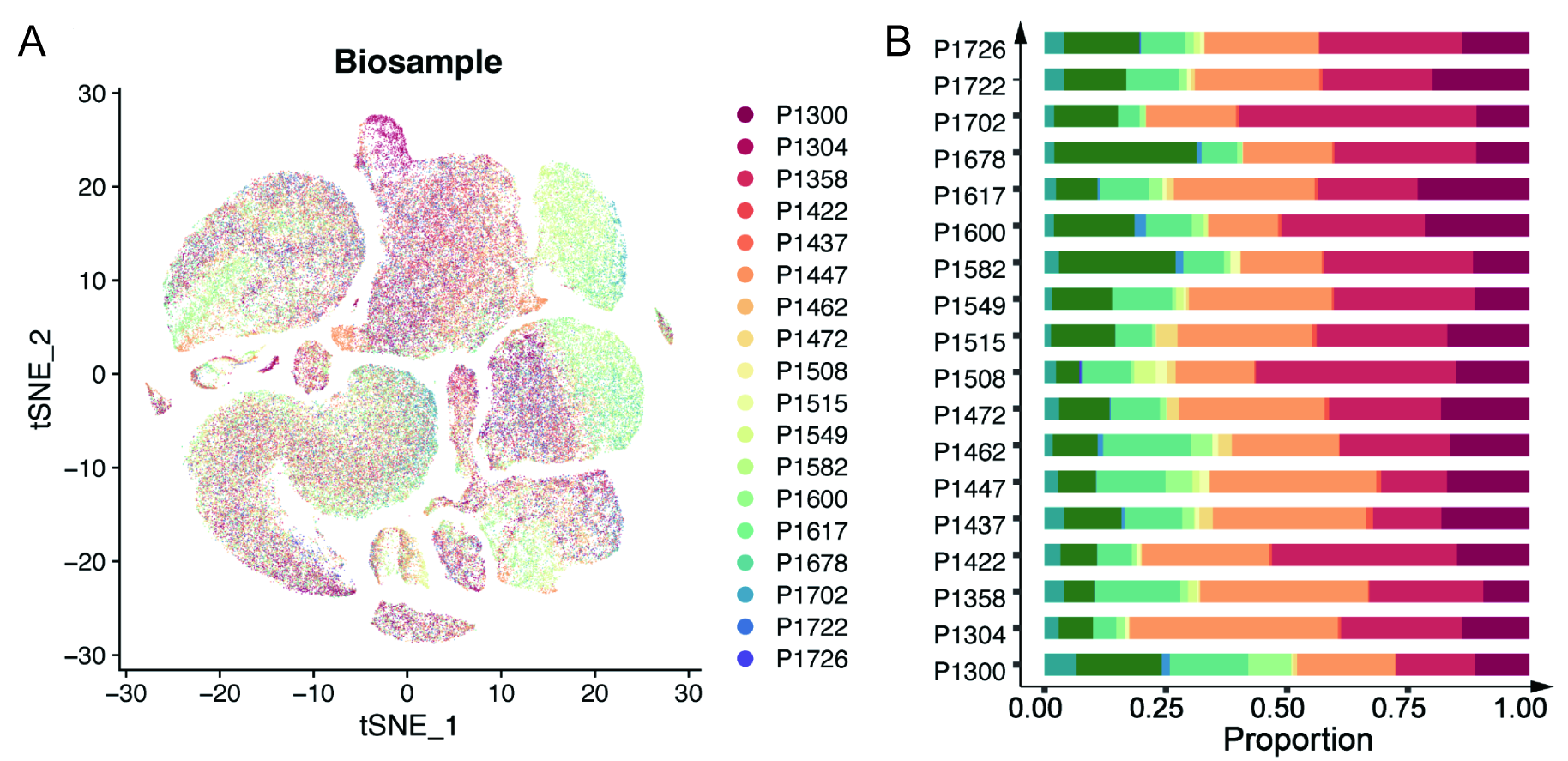

Supplement: Supplementary file 1 [file DataSheet1.zip › revised supplementary figures part 1/Figure S1.tif]

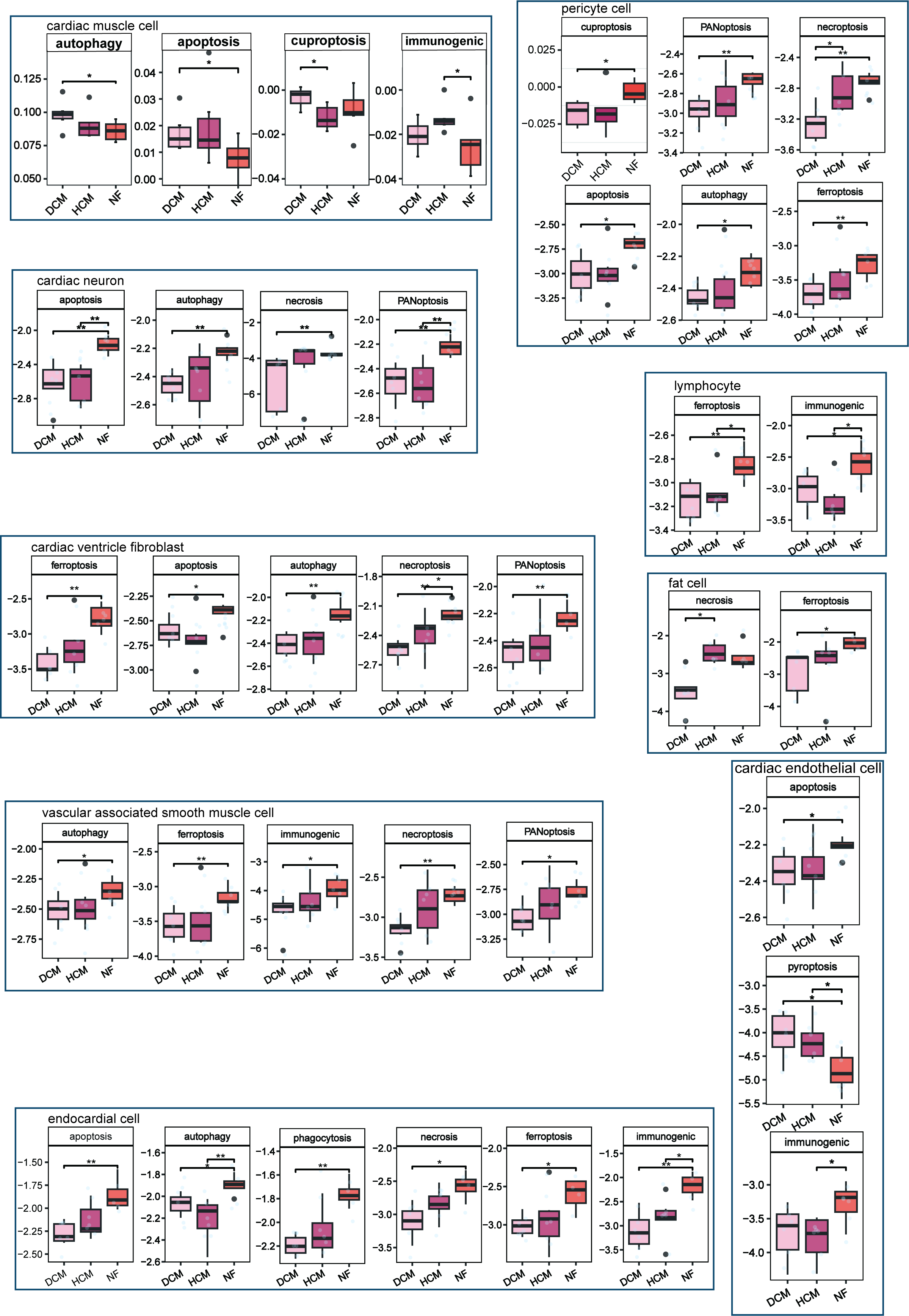

Supplement: Supplementary file 1 [file DataSheet1.zip › revised supplementary figures part 1/Figure S2.tif]

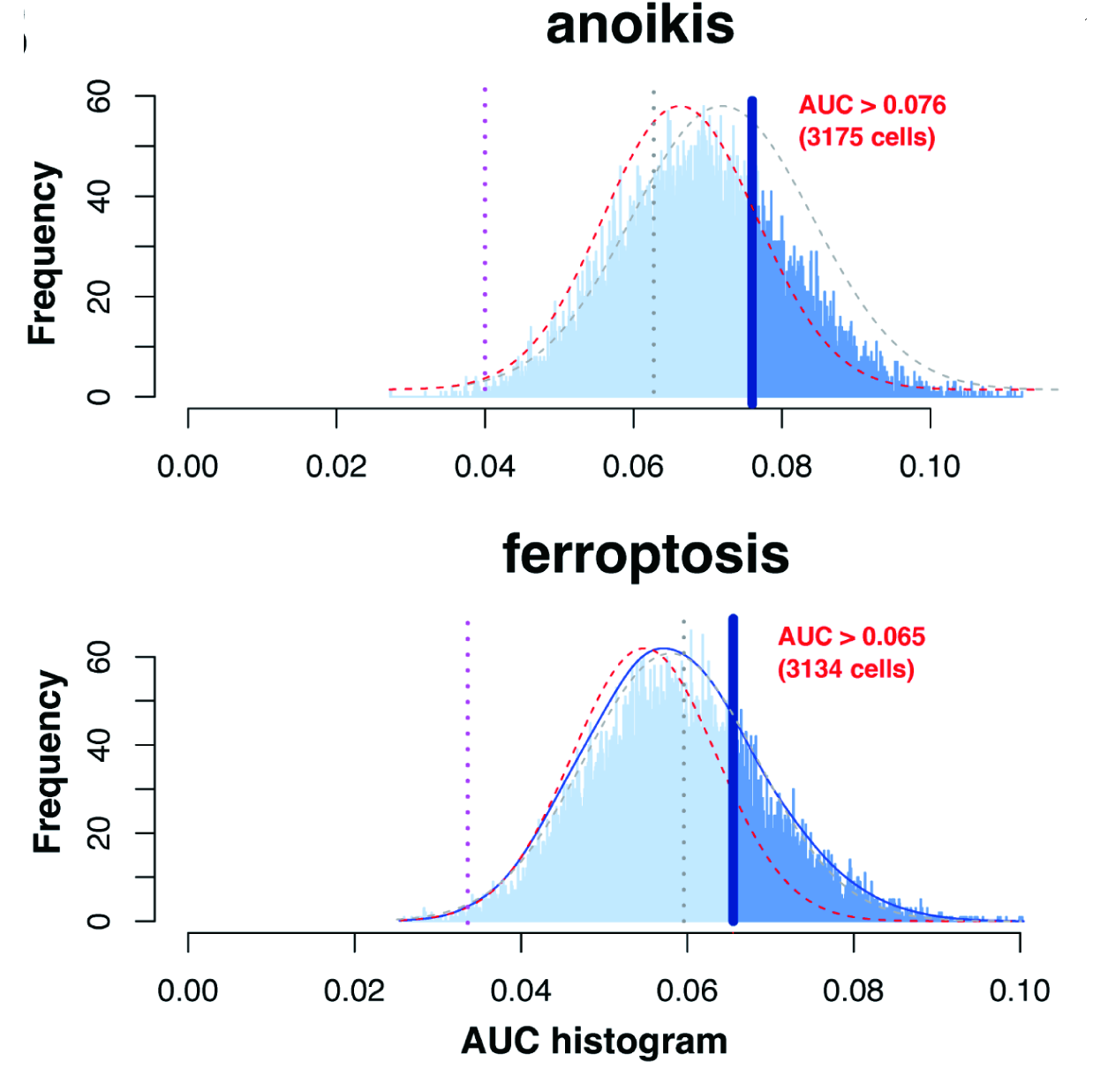

Supplement: Supplementary file 1 [file DataSheet1.zip › revised supplementary figures part 1/Figure S3.tif]

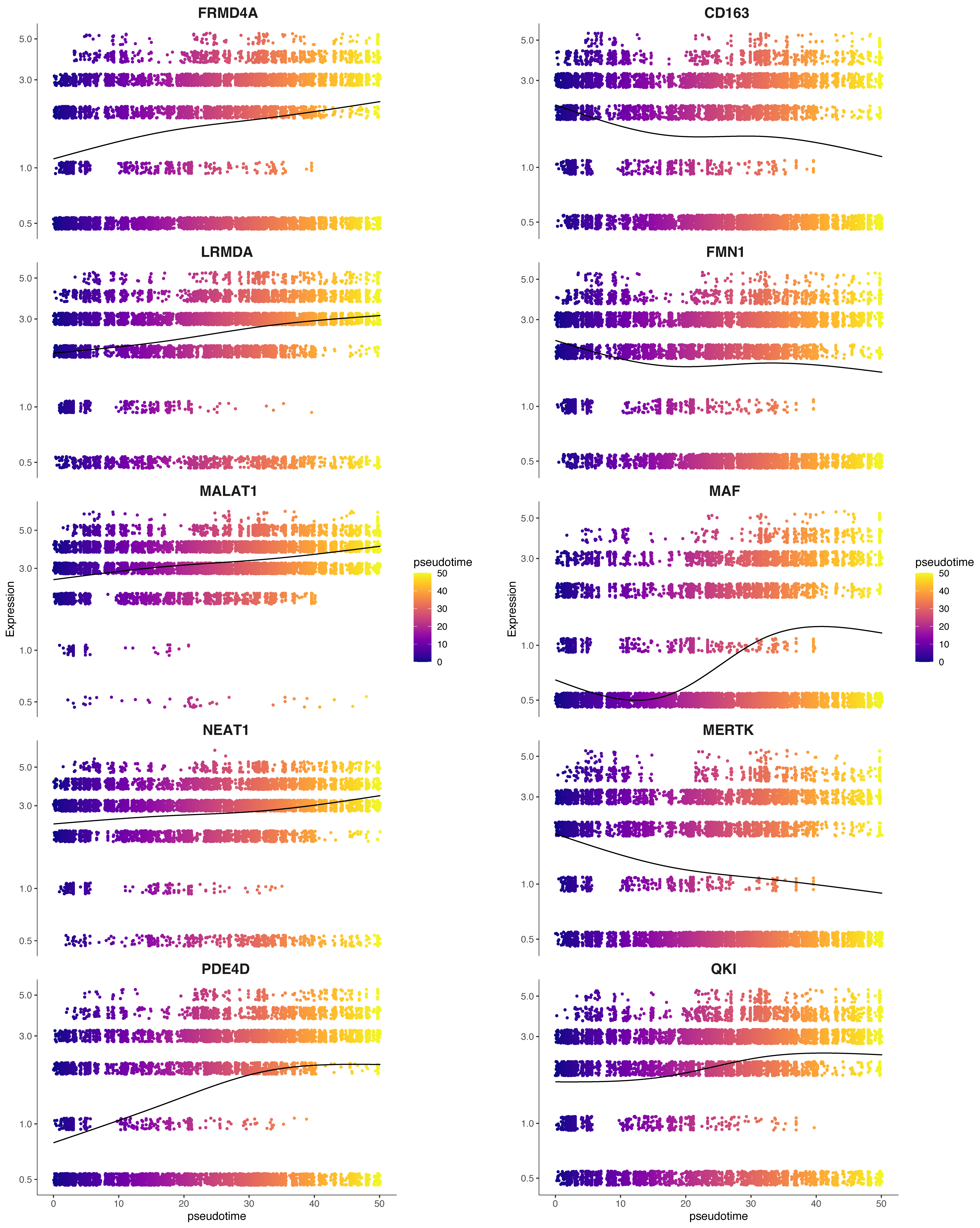

Supplement: Supplementary file 1 [file DataSheet1.zip › revised supplementary figures part 1/Figure S4.tif]

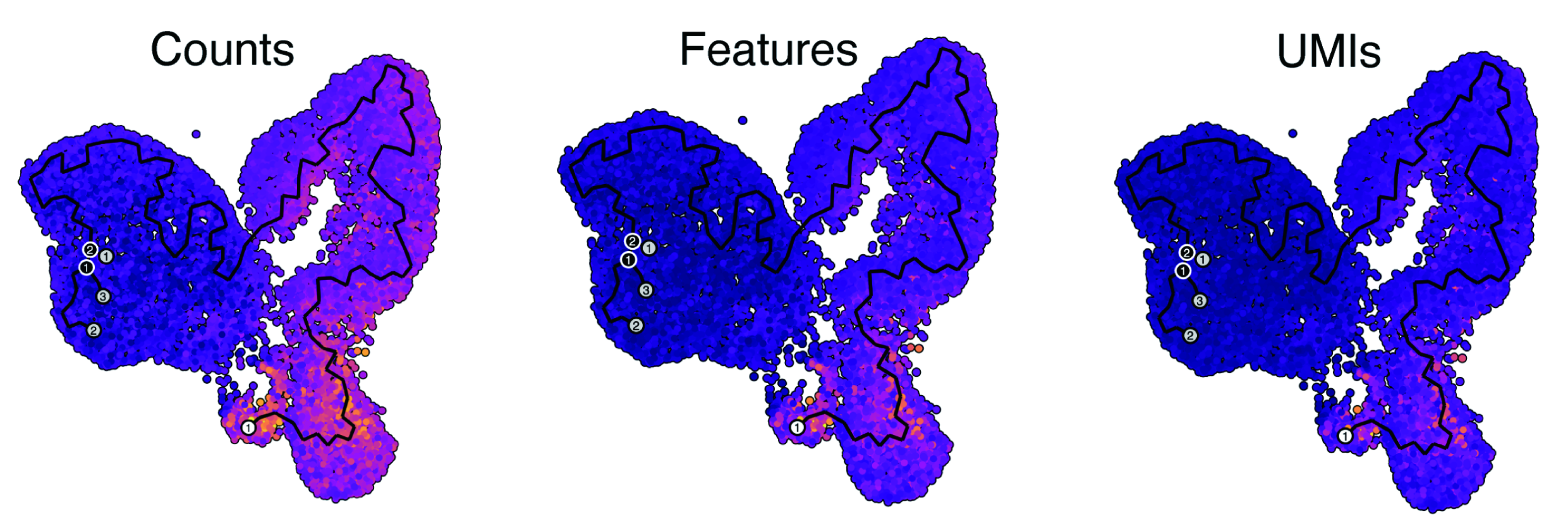

Supplement: Supplementary file 1 [file DataSheet1.zip › revised supplementary figures part 1/Figure S5.tif]

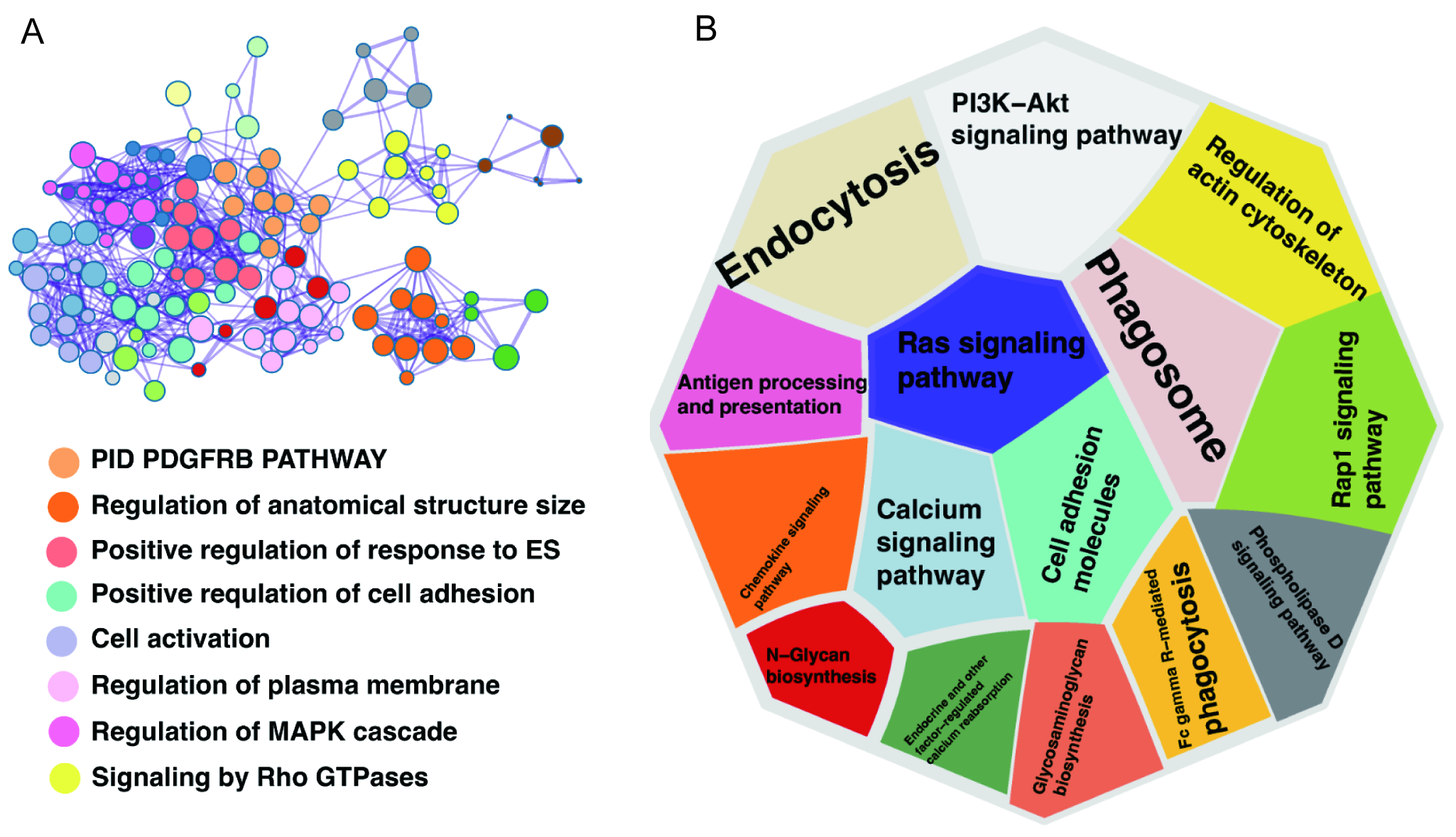

Supplement: Supplementary file 1 [file DataSheet1.zip › revised supplementary figures part 1/Figure S6.tif]

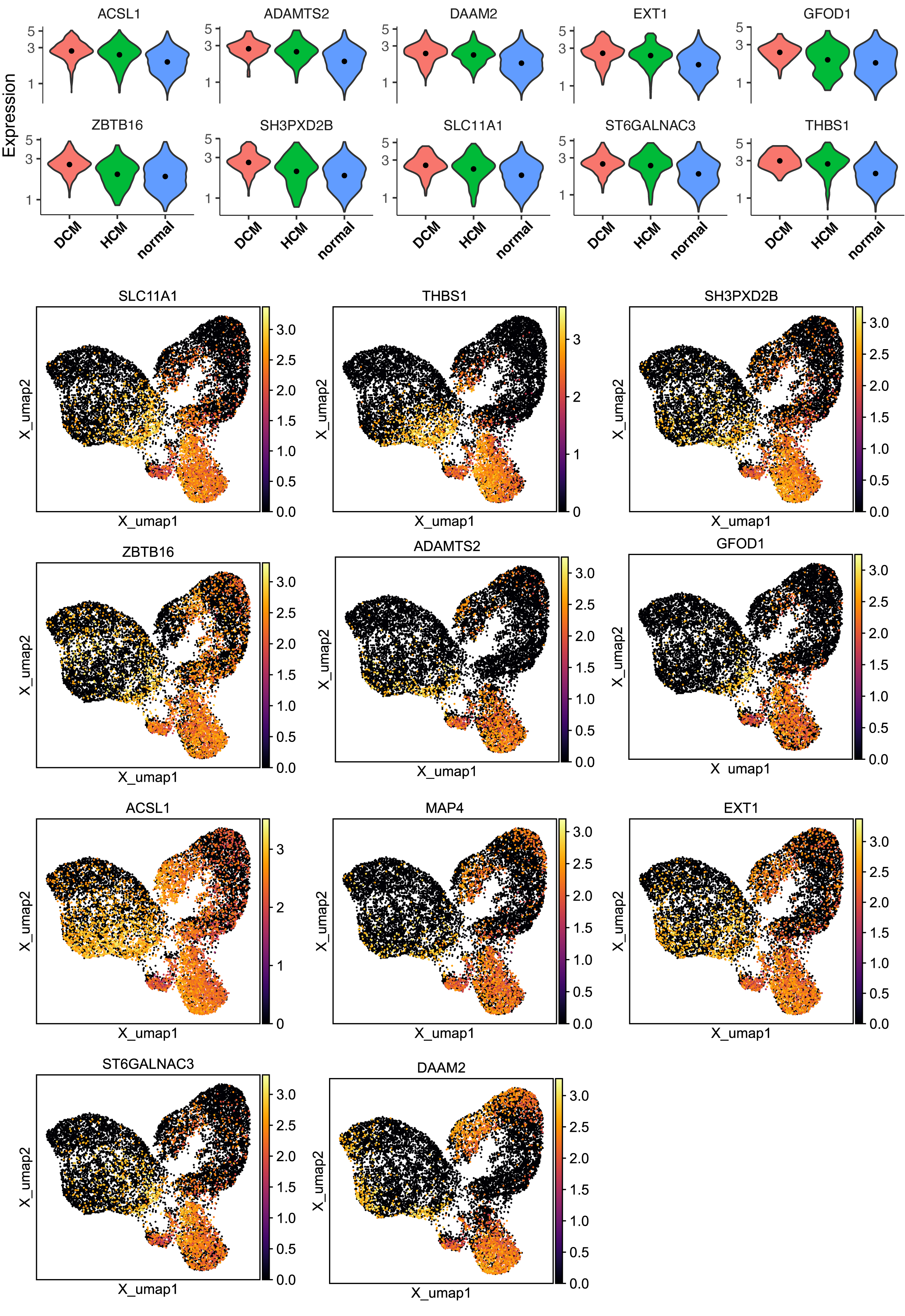

Supplement: Supplementary file 1 [file DataSheet1.zip › revised supplementary figures part 1/Figure S7.tif]

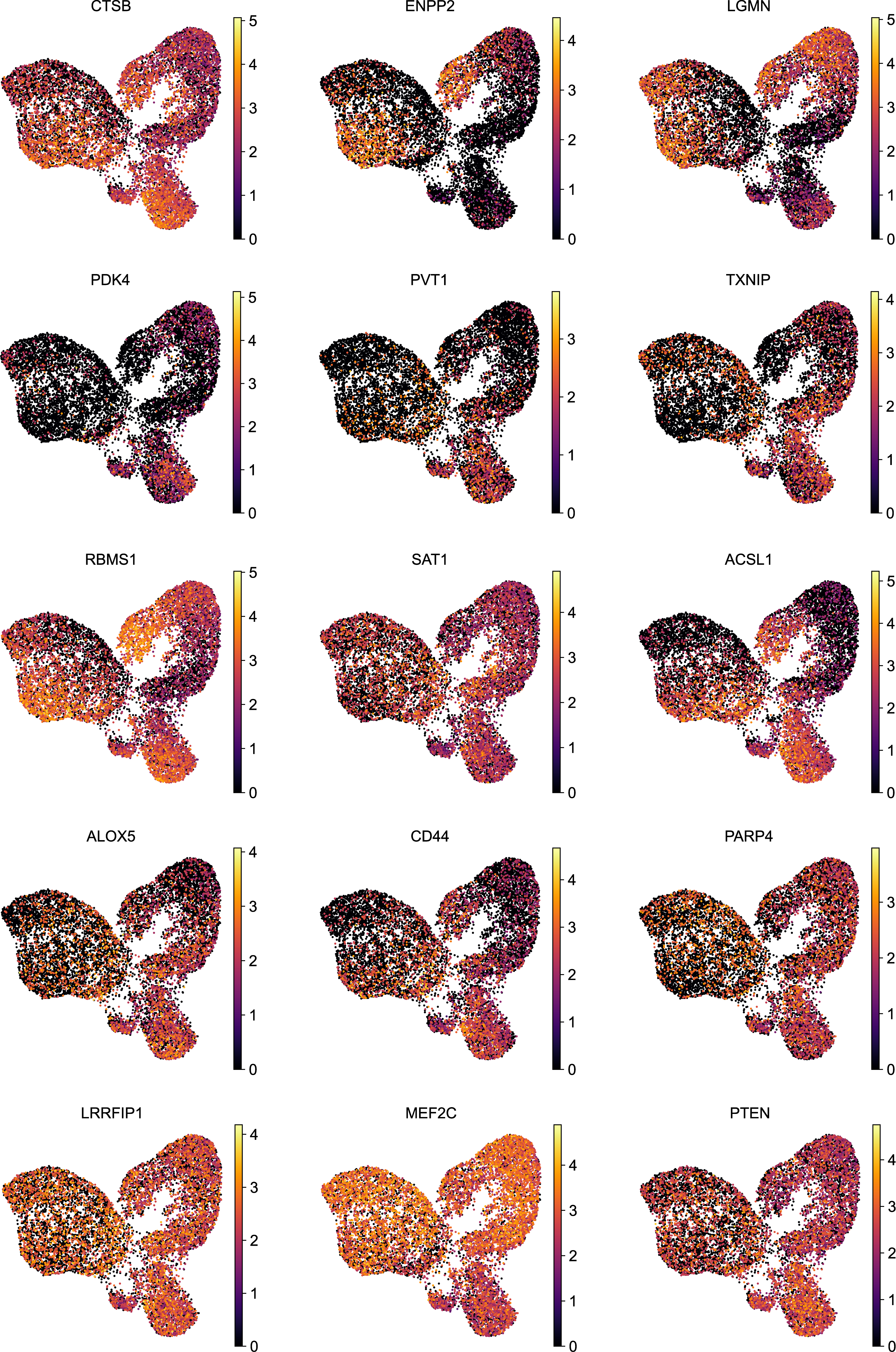

Supplement: Supplementary file 1 [file DataSheet1.zip › revised supplementary figures part 1/Figure S8.tif]

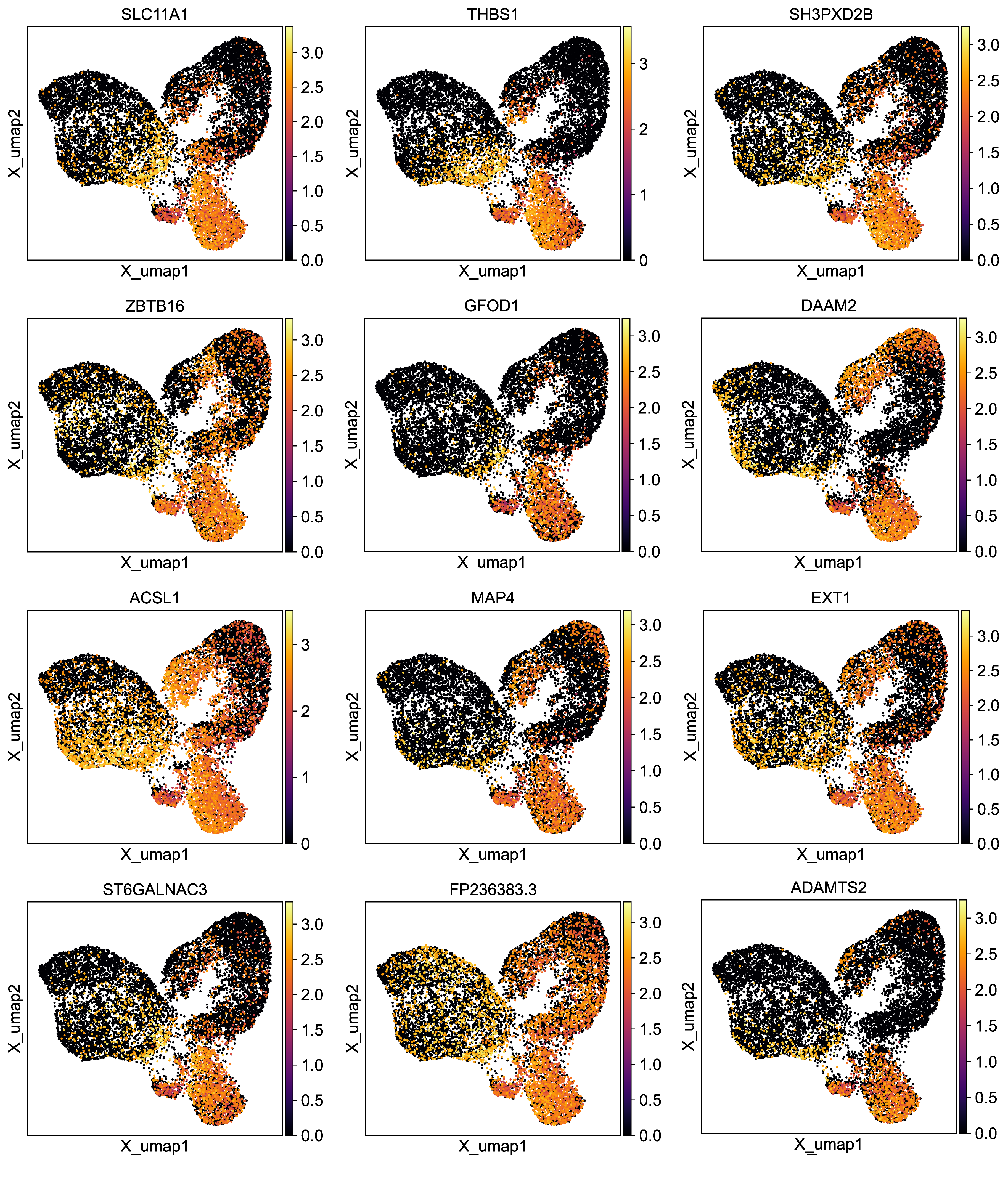

Supplement: Supplementary file 2 [file DataSheet2.zip › revised supplementary figures part 2/Figure S10.tif]

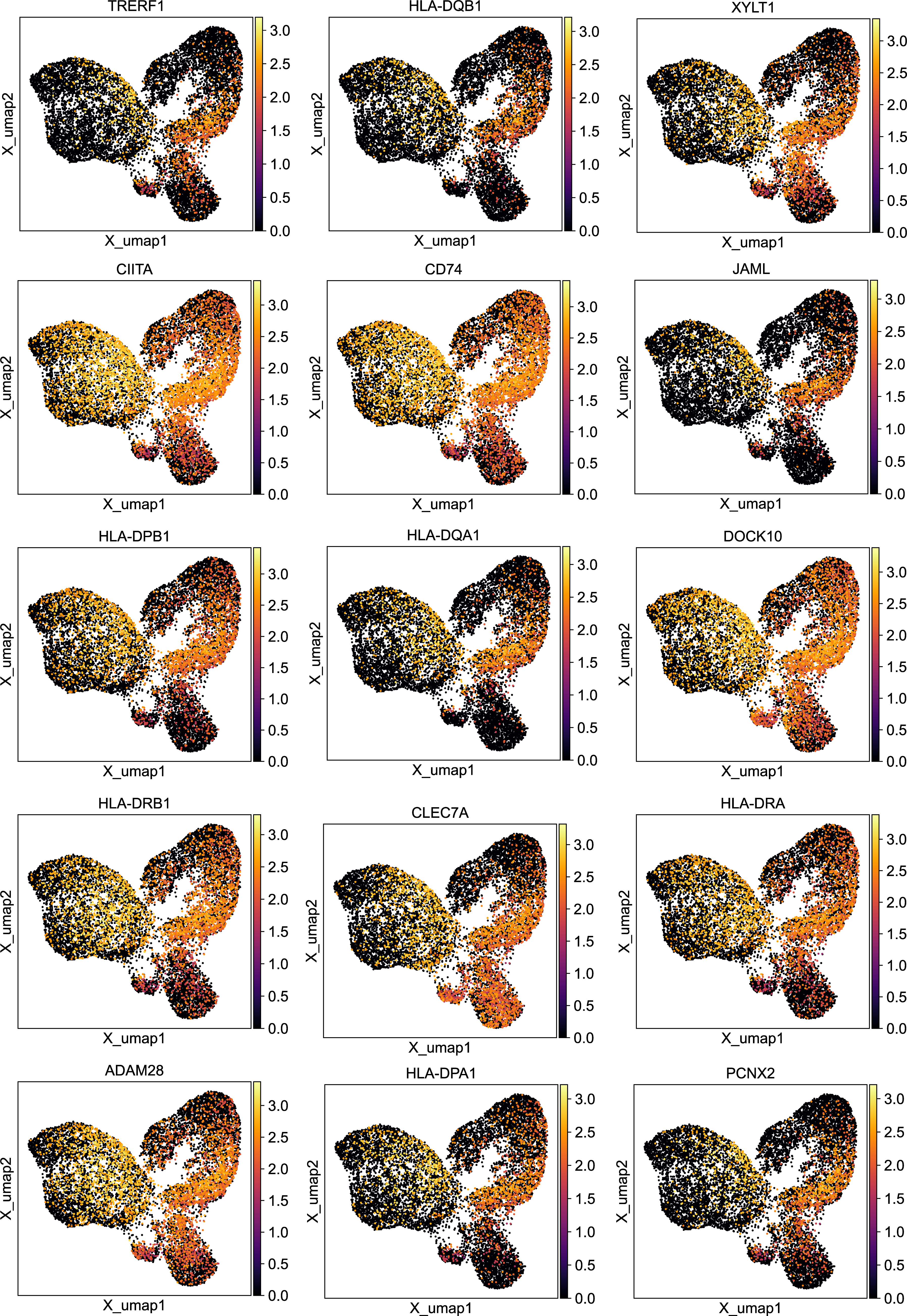

Supplement: Supplementary file 2 [file DataSheet2.zip › revised supplementary figures part 2/Figure S11.tif]

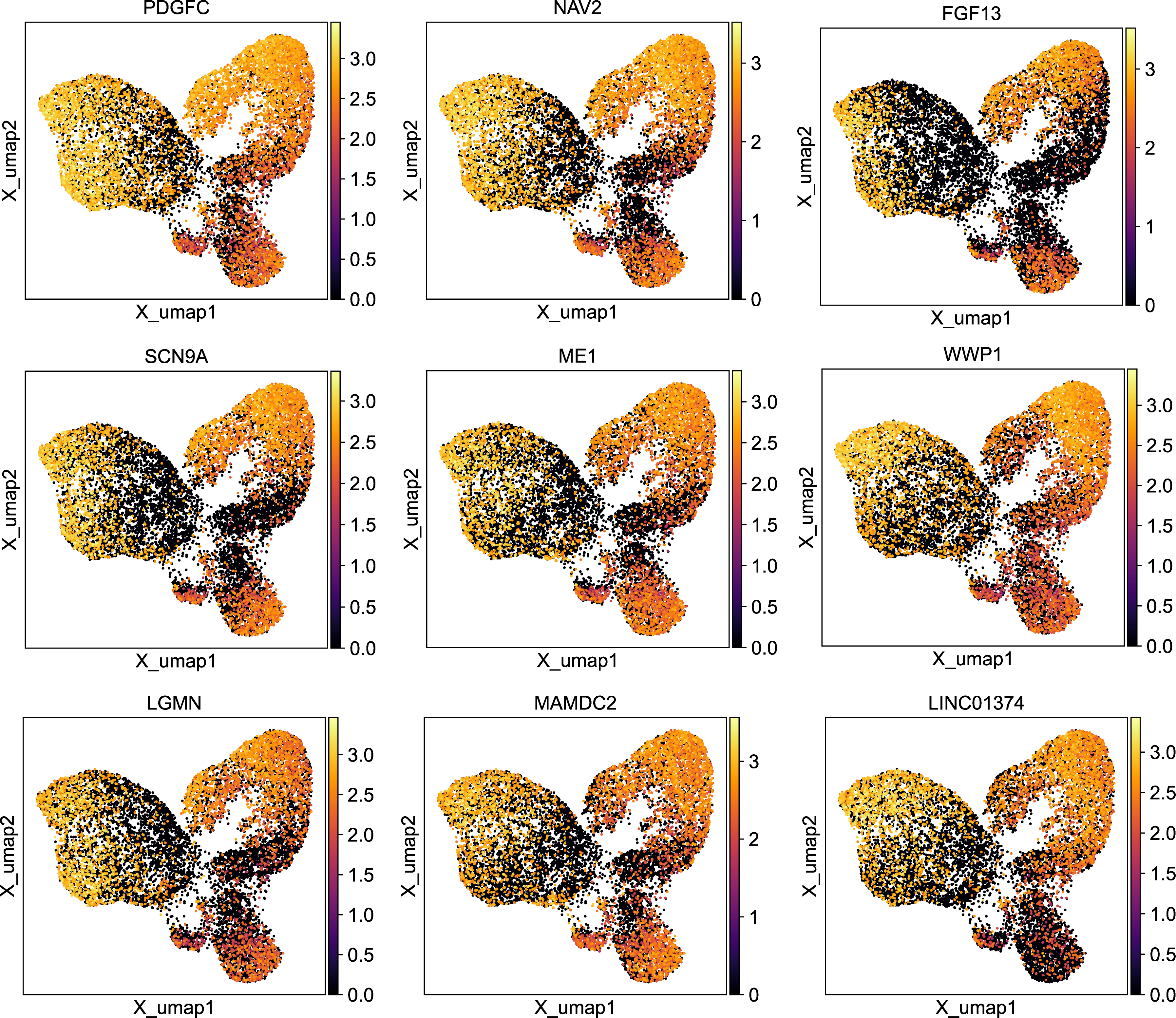

Supplement: Supplementary file 2 [file DataSheet2.zip › revised supplementary figures part 2/Figure S12.tif]

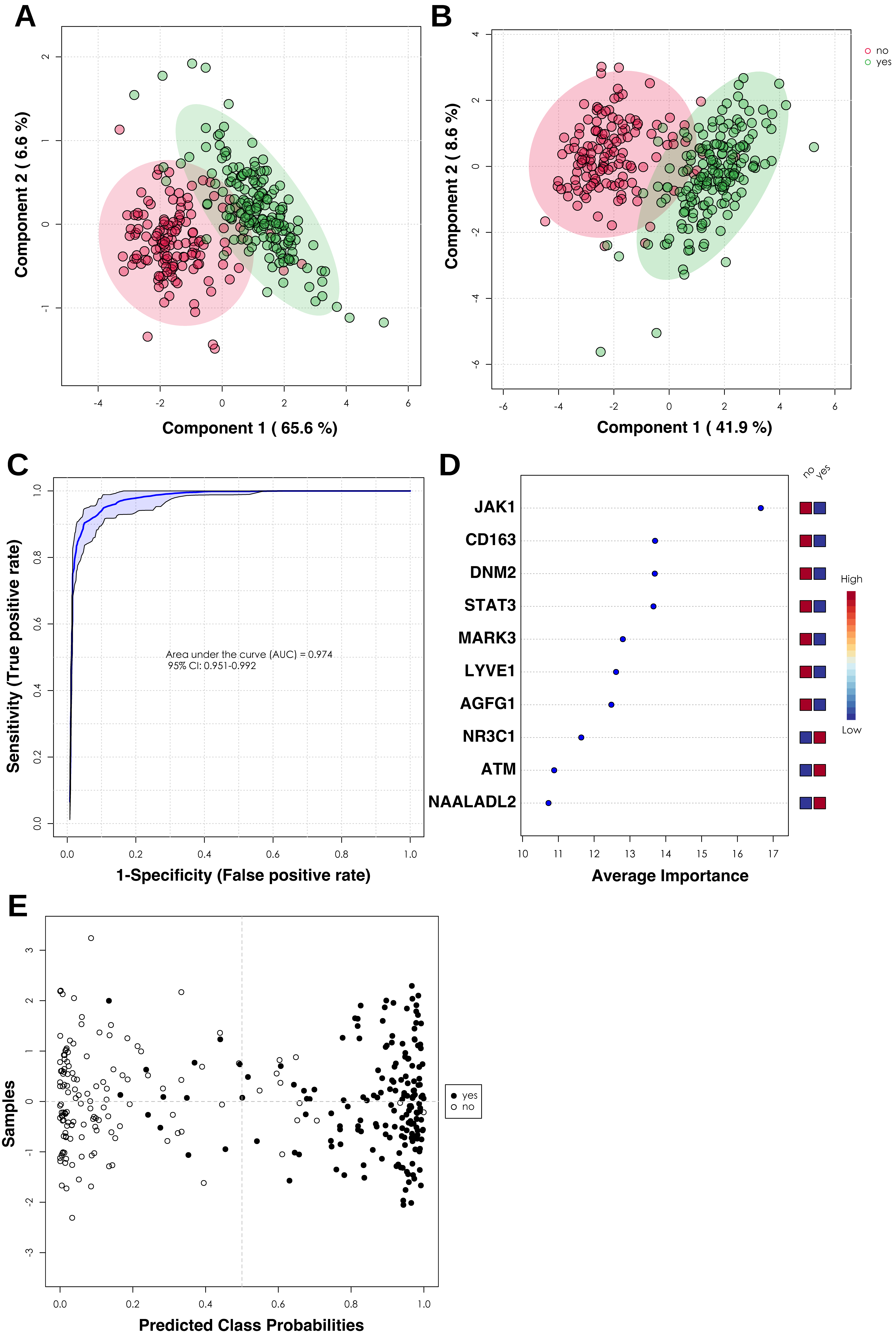

Supplement: Supplementary file 2 [file DataSheet2.zip › revised supplementary figures part 2/Figure S13.tif]

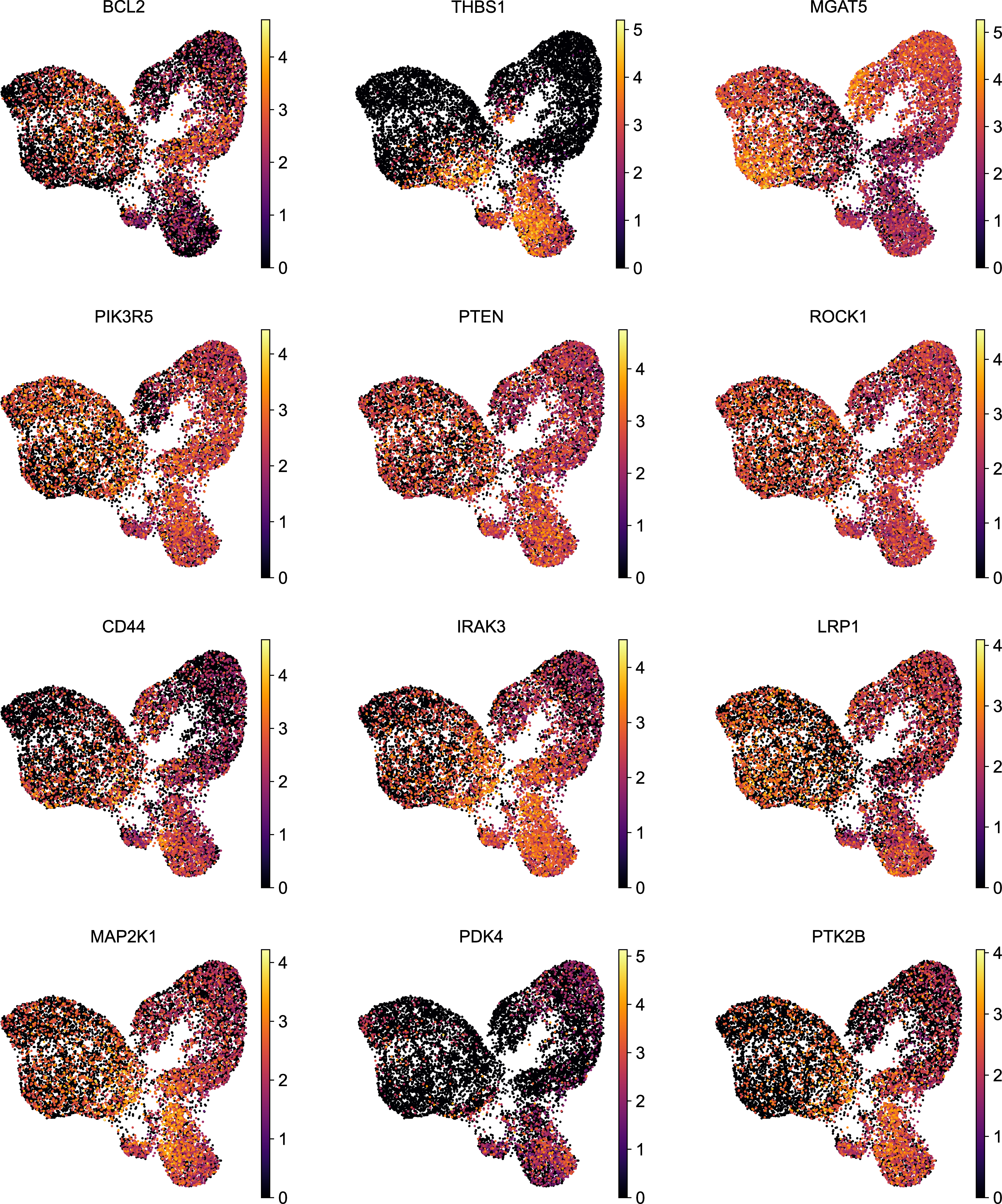

Supplement: Supplementary file 2 [file DataSheet2.zip › revised supplementary figures part 2/Figure S9.tif]
