# Supplementary material for: Deciphering macrophage differentiation and cell death dynamics in heart failure: a single-cell sequencing odyssey
Source: Front Immunol. 2025 Oct 7;16:1604226. doi: 10.3389/fimmu.2025.1604226 (PMC12537381; doi:10.3389/fimmu.2025.1604226)
Supplement: Supplementary file 2 [file DataSheet2.zip › revised supplementary figures part 2/Supplementary figure legends.docx]

**Supplementary figure legends**

**Figure S1.** Additional sample‑ and composition‑level views from Figure 1. A. t‑SNE plot of 18 individual patient samples, with each color corresponding to a unique biosample identifier (patient ID). B. Proportions of each cell type across all samples, using the same color scheme as Figure 1A.

**Figure S2. ssGSEA‑based activity scores of cell death–related pathways across different cardiac cell types and disease severities. Box plots show normalized enrichment scores for the relevant pathways in each cell type. Samples are grouped by clinical status (NF, DCM, HCM), with statistical significance indicated by asterisks (* *p*< 0.05; ** *p* < 0.01).**

**Figure S3.** Thresholds defining activation states for cell death programs. Threshold values for determining activation states of anoikis (AUC > 0.076) and ferroptosis (AUC > 0.065) based on AUCell scores.

**Figure S4.** Genes most strongly correlated with pseudotime in macrophages and their expression dynamics along the inferred trajectory. Each panel shows single‑cell expression values for one representative gene (e.g., FRMD4A, CD163) plotted against pseudotime. Points are colored by pseudotime (continuous gradient from dark purple [early] to bright yellow [late]), illustrating temporal changes in expression during macrophage differentiation.

**Figure S5. Per‑cell quality metrics along the pseudotime trajectory, showing counts, features, and Unique Molecular Identifier (UMI) counts for cells positioned along the inferred trajectory. Counts represent the total number of transcripts (sequencing reads) detected per cell, reflecting sequencing depth and capture efficiency; features indicate the number of distinct genes detected per cell, reflecting transcriptomic complexity; and UMIs (Unique Molecular Identifiers) represent the number of unique transcript molecules per cell after removing PCR duplicates, providing a more accurate quantification of true molecular counts. In each panel, the color gradient from dark purple (low) to bright yellow (high) represents the relative value of the corresponding metric for each cell. Marker ① denotes the common root state (trajectory starting point), from which cells diverge along distinct pseudotime branches, while markers ② and ③indicate alternative terminal states (endpoints) reached via different differentiation paths. The presence of multiple paths (e.g., ① → ② and ① → ③) reflects branching in the trajectory, suggesting that cells originating from the same early state can adopt divergent fates, potentially corresponding to distinct functional phenotypes or disease‑associated states.**

**Figure S6.** Regression‑ and enrichment‑based analysis of pseudotime‑associated genes. A. Regression analysis of pseudotime and gene expression levels. B. Enrichment analysis of genes from regression analysis.

**Figure S7.** Expression profiles and spatial distribution of significantly downregulated key genes in diseased cardiac tissues. The upper panels show violin plots depicting normalized expression levels of each gene across major cardiac cell types. The lower panels present UMAP projections of single cells, colored by normalized expression (black = low, orange = moderate, bright yellow = high), illustrating the spatial distribution of these genes within the cellular landscape.

**Figure S8.** Spatial expression patterns of differentially expressed ferroptosis‑related genes in healthy versus diseased cardiac tissues. Each panel shows the distribution of a representative gene (e.g., *CTSB*, *ENPP2*) across the cellular embedding. Color gradients indicate normalized expression levels (dark colors = low, light colors = high), illustrating differences in expression between healthy and diseased groups.

**Figure S9.** Spatial expression patterns of differentially expressed anoikis‑related genes in healthy versus diseased cardiac tissues. Each panel displays the distribution of a representative gene (*BCL2*, *THBS1*) across the cellular embedding. Color gradients indicate normalized expression levels (dark colors = low, light colors = high), highlighting differences in expression between healthy and diseased groups.

**Figure S10.** UMAP visualization of Macrophage‑1–specific marker genes. Each panel shows the spatial distribution of a representative marker gene across the Macrophage‑1 population. Cells are colored by normalized expression level, with darker shades indicating lower expression and lighter shades indicating higher expression, illustrating the localization and relative abundance of each marker within the embedding.

**Figure S11.** UMAP visualization of Macrophage‑2–specific marker genes. Each panel shows the spatial distribution of a representative marker gene across the Macrophage‑2 population. Cells are colored by normalized expression level, with a continuous gradient from purple (low expression) to yellow (high expression), illustrating the localization and relative abundance of each marker within the embedding.

**Figure S12.** UMAP visualization of Macrophage‑3–specific marker genes. Each panel shows the spatial distribution of a representative marker gene across the Macrophage‑3 population. Cells are colored by normalized expression level, with a continuous gradient from black (low expression) through red/orange to yellow (high expression), illustrating the localization and relative abundance of each marker within the embedding.

**Figure S13.** Performance evaluation of the multigenic model for heart failure using different feature sets. A–B. Principal component analysis (PCA) plots based on the top 20 genes selected for maximal group separation, illustrating the distribution of heart failure and control samples in the first two principal components. Each point represents an individual sample, colored by group. The proportion of variance explained by PC1 and PC2 is shown on the respective axes. A larger feature set (top 20 genes) was used here to better capture overall variance and enhance visualization of sample separation. C. Receiver operating characteristic (ROC) curve of the classification model, with the area under the curve (AUC) value and its 95% confidence interval , shown as the shaded region.D. Ranked importance of the top 10 predictive genes in the model. The x‑axis shows the average importance score, with genes ordered from most to least important. Point colors indicate the relative expression level of each gene within the “yes” group (heart failure samples), with red representing higher expression and blue representing lower expression in that group
